# Multi-Source Data Fusion Integrating HPLC, GC-IMS, and Multidimensional Biomimetic Technologies for Evaluating the Effects of Different Processing Degrees on the Quality of Salt-Processed *Semen Cuscutae*

**DOI:** 10.3390/foods15193570

**Published:** 2026-10-08

**Authors:** Chenyang Deng, Mengqing Liu, Kaiwen Chen, Cetao Tang, Yue Kang, Hao Zhang, Yazhong Zhang, Chuanshan Jin, Deling Wu, Xiaoli Wang

**Affiliations:** 1 Department of Pharmacy, Anhui University of Chinese Medicine, Hefei 230012, Chinadlwu7375@ahtcm.edu.cn (D.W.); 2 The Anhui Institute for Food and Drug Control, Hefei 230051, China; 3 Heritage Base of TCM Processing Technology of NATCM, Anhui University of Chinese Medicine, Hefei 230012, China; 4 Anhui Province Key Laboratory of Traditional Chinese Medicine Decoction Pieces of New Manufacturing Technology, Hefei 230012, China; 5 School of Chinese Medicine and Health, Bozhou University, Bozhou 236800, China

**Keywords:** *Semen Cuscutae*, processing degrees, AHP-TOPSIS, electronic nose

## Abstract

*Semen Cuscutae* is commonly used as a medicinal and edible functional food ingredient. Salt-processing can significantly alter its sensory quality and endogenous components. Currently, salt-processed *Semen Cuscutae* mainly adopts hyperoside as the quality evaluation indicator, which fails to meet the requirements for the identification of processing intensity (heat level) during the development of the traditional Chinese medicine industry. This study aims to construct a comprehensive quality evaluation framework for identifying the optimal processing degree of salt-processed *Semen Cuscutae*. Multi-dimensional bionic technologies including machine vision, electronic nose and electronic tongue, combined with HPLC and GC-IMS, were applied to analyze the changes in sensory characteristics and endogenous components of salt-processed *Semen Cuscutae* across different processing degrees. Characteristic indicators were comprehensively scored using the AHP-TOPSIS method. Compared with other groups, appropriately processed salt-processed *Semen Cuscutae* exhibited a brown appearance, significantly different W1W/W2W sensor responses, resulting in a more harmonious taste profile and distinct volatile chemical markers. Further correlation analysis was performed among bionic sensing indicators, endogenous components and GC-IMS-derived volatile data. AHP-TOPSIS results demonstrated that salt-processed *Semen Cuscutae* with an appropriate processing degree exhibits optimal comprehensive quality. The comprehensive quality evaluation framework established in this study provides scientific support for the process control and quality assessment of *Semen Cuscutae* as a functional food ingredient, and also offers a methodological reference for the processing evaluation of similar medicinal and edible food materials.

## 1. Introduction

*Semen Cuscutae* refers to the dried mature seed of either *Cuscuta australis* R. Br. or *Cuscuta chinensis* Lam. It is a commonly used ingredient in traditional Chinese dietary therapy and functional foods. These two species both belong to the Convolvulaceae family [1]. Modern studies have shown that *Semen Cuscutae* possesses various bioactivities, including immune enhancement, reproductive function improvement, anti-aging, and liver protection. Its material basis primarily includes flavonoids, along with polysaccharides, alkaloids, and other components. Its unique composition of active ingredients makes it a high-quality raw material for the development of functional foods. Consequently, it has garnered significant attention in food and functional product research [2].

Raw *Semen Cuscutae* has a grayish-brown seed coat and a firm texture. After heating and stir-frying, it turns brownish-yellow and releases an aroma, accompanied by cracked seed coats that facilitate the dissolution of active components during decoction. *Semen Cuscutae* moistened with salt solution is documented in the Chinese Pharmacopoeia (2025 Edition). Salt-processed *Semen Cuscutae* exhibits a more descending medicinal nature, according to the principle that “salt-processing guides the action to the kidney meridian” [3]. Salt-processing enhances the delivery of active components to the kidney meridian and strengthens the therapeutic effects of tonifying the kidneys and consolidating essence [4]. Meanwhile, this processing alters the contents of endogenous active components such as hyperoside and astragalin [5]. Its flavor transforms from the original woody and pungent notes to roasted and ester-like aromas, forming the characteristic profile of “slightly aromatic in odor, slightly salty in taste.” For food processing, this transformation of flavor and active ingredients is the crucial core for optimizing food sensory quality and ensuring the stable delivery of health-promoting effects, directly affecting the taste experience and product quality uniformity of functional foods. However, the judgment of the traditional processing endpoint has long depended on sensory experience such as visual inspection and manual palpation, leading to high subjectivity and unstable product quality. Traditional single-marker quantitative methods also have limitations in reflecting the overall quality of salt-processed *Semen Cuscutae*, as they ignore volatile components and taste-active compounds. It is difficult to meet the core requirements of unified quality control in modern food industrialization, greatly limiting the industrialization of *Semen Cuscutae*-based functional foods. Currently, many scholars have combined bionic sensing technologies including the electronic nose and electronic tongue, alongside instrumental analysis such as gas chromatography–ion mobility spectrometry (GC-IMS). These technologies have been applied for authenticity identification of *Bletilla striata* decoction pieces, as well as quality discrimination and geographical origin traceability of medicinal materials such as *Astragalus membranaceus*.

Traditional quality identification mainly relies on sensory evaluation (sight, smell, taste, touch), microscopic observation, and simple physicochemical tests. These methods are intuitive and rapid. However, they are highly subjective and difficult to quantify, failing to meet the requirements of accurate and objective quality evaluation demanded by the modern food industry. To address this issue, machine vision and analytical technologies have been developed, aiming to achieve more objective and quantifiable integrated quality evaluation. This technical system mainly includes two categories: bionic sensing and precision instrumental analysis. Bionic sensing technologies, such as the electronic nose [6], generate the “odor fingerprint” of samples. The electronic tongue captures the “taste fingerprint” [7], while machine vision extracts digital appearance features such as color (RGB values) [8]. Precision instrumental analysis, such as high-performance liquid chromatography (HPLC), serves as the gold standard for separating and quantifying bioactive compounds. Gas chromatography–ion mobility spectrometry (GC-IMS) enables simultaneous identification of volatile components. They provide a foundation for more objective and quantifiable quality control by converting sensory information into objective, quantifiable digital signals. Multi-source information fusion further integrates these objective datasets with molecular-level information acquired via precision instruments to build a more robust evaluation system.

In summary, this study aims to construct a comprehensive, objective, and digital strategy to correlate and screen multi-dimensional processing data of salt-processed *Semen Cuscutae*. We integrate RGB image analysis, an electronic nose, an electronic tongue and HPLC, and innovatively incorporate gas chromatography–ion mobility spectrometry (GC-IMS) [9]. The core strategy adopts multivariate statistical methods to perform dimensionality reduction on multi-source datasets and screen critical characteristic markers. Finally, the AHP-TOPSIS method is introduced to assign weights to quantitative indicators from different dimensions, so as to establish an objective quality evaluation model capable of comprehensively discriminating samples with different processing degrees. This work intends to provide scientific and efficient technical support for the standardization of stir-frying procedures and the consistency control of product quality for *Semen Cuscutae* as a functional food ingredient, and also to offer a methodological reference for the processing evaluation of similar medicinal and edible food materials.

## 2. Materials and Methods

### 2.1. Materials and Instruments

Fifteen batches of raw *Semen Cuscutae* were purchased from the Bozhou Traditional Chinese Medicine Professional Trading Market (Bozhou, China), and all were identified by Associate Professor Yang Qingshan from Anhui University of Chinese Medicine as the dried mature seeds of *Cuscuta australis* R.Br. (Convolvulaceae). These 15 independent raw material batches were harvested from three major producing regions of *Semen Cuscutae* in China, including Inner Mongolia, Liaoning, and Ningxia, covering the majority of the country’s primary cultivation areas. Complete batch numbers and origin information are documented in Supplementary Table S8. The production year was 2023. The moisture content of the raw material before processing was (7.5 ± 0.5)%. All samples were sealed and stored in a cool, dry place, protected from direct sunlight and moisture. For experimental analysis, the 15 raw material batches were independently stir-fried, covering four different processing degrees, generating a total of 60 independent processing batch samples. The reference standards of hyperoside (DSTDB001263), astragalin (DSTDB038623), isoquercitrin (DSTDB001752), quercetin (DSTDB004857), and kaempferol (DSTDB002431) were all purchased from Chengdu (Sichuan, China), Lemaitian Pharmaceutical Technology Co., Ltd., with purities ≥ 98%. Salt (CNSG Dongxing Salt Chemical Co., Ltd, Dongxing, Guangxi, China) MC-CLE3367; Normal ketones C4–C9 (Analytical Reagent) (Aladdin, Shanghai, China); Nitrogen gas (99.999%) (Shanghai Yuanye Bio-Technology Co., Ltd. Shanghai, China); 2-Octanol (Shanghai Yuanye Bio-Technology Co., Ltd, Shanghai, China); IH Midea cooking stove: Guangdong Midea Life Electric Appliance Manufacturing Co., Ltd, Foshan, Guangdong, China; CN-LQC21001 electronic balance: Kunshan Youkeweite Electronic Technology Co., Ltd.; Kunshan, Jiangsu, China. FA2004 analytical balance: Shanghai Jingke Balance, Shanghai, China. Alpha 7C II camera: Sony Corporation Tokyo, Japan; FW-100 high-speed multifunctional crusher: Beijing Yongguangming Medical Instrument Co., Ltd., Beijing, China; FlavourSpec^®^ gas chromatography–ion mobility spectrometer: G.A.S, Dortmund, Germany. TS-5000Z electronic tongue (Insent Inc., Atsugi, Japan); the sensor array is detailed in Table S2. UltiMate 3000 system equipped with a UV detector (Thermo Fisher Scientific, Waltham, MA, USA). CTC-PAL 3 static headspace autosampler (CTC Analytics AG, Zwingen, Switzerland); VOCal data processing software (0.4.10: G.A.S., Dortmund, Germany), MXT-WAX capillary column (15 m × 0.53 mm, 1.0 μm, Restek, Bellefonte, PA, USA). EnSight multimode microplate reader (PerkinElmer, Waltham, MA, USA); transparent 96-well microplates; thermostatic water bath; high-speed centrifuge.

### 2.2. Preparation of Salt-Processed Semen Cuscutae with Different Processing Degrees

Salt-processed *Semen Cuscutae* with different processing degrees were prepared, including the raw products (S), under-processed products (BG), appropriately processed (SZ), and over-processed (TG) products. A 600 W Midea induction cooker (Guangdong Midea Life Electric Manufacturing Co., Ltd., Foshan, China) was used for stir-frying, together with a standard stainless steel wok (upper inner diameter 22 cm, depth 8 cm, body thickness 2 mm). The wok was preheated for 1 min before sample loading. Real-time temperature data were collected by thermocouples at 30 s intervals. The temperature was maintained at 180 ± 5 °C during the whole heating process under continuous uniform stirring (approximately 40 times/min) to achieve homogeneous heating of samples. A total of 15 batches of raw *Semen Cuscutae* were used. For each treatment batch, 100 g of raw *Semen Cuscutae* decoction pieces were stir-fried for 2 min, 4 min, and 7 min, respectively. Samples of each group were stir-fried sequentially in the order of BG, SZ, and TG, with each batch operated independently, yielding three groups of samples: under-processed, appropriately processed, and over-processed. The moisture content before processing was (9.0 ± 0.5)%. After stir-frying, the samples were naturally cooled at room temperature.

#### 2.2.1. Preparation of Raw Products

According to the 2025 edition of the Chinese Pharmacopoeia [10], purified *Semen Cuscutae* were selected, and impurities were removed. And the seeds were washed and dried to obtain the raw *Semen Cuscutae* pieces. Then, 100 g was weighed.

#### 2.2.2. Saline Moistening and Steeping

According to the salt-processing method for “*Semen Cuscutae*” in the 2025 edition of the Chinese Pharmacopoeia [10], 100 g of the raw *Semen Cuscutae* was taken, and salt was weighed in a sample-to-salt ratio of 50:1. Purified water (approximately 5 times the weight of the salt) was used to prepare the saline solution. The seeds were moistened and steeped in this saline solution for 30 min. With the cooking stove set at a power of 600 W, the moistened and steeped *Semen Cuscutae* were then placed into the pot.

#### 2.2.3. Preparation of the Under-Processed Sample

Preheat the wok to the specified temperature, add the moistened and steeped medicinal material, and stir-fry for 2 min. Stir-fry until the surface of the sliced pieces turns light brown, slightly cracked, and emits a faint aroma. Quickly remove from the wok, spread evenly to cool, and obtain the under-processed salt-processed *Semen Cuscutae* sample.

#### 2.2.4. Preparation of the Appropriately Processed Sample

Stir-fry for 4 min until the surface of the herbal pieces turns yellow-brown, cracks, and emits a faint aroma. Quickly remove from the wok, spread evenly to cool, and obtain the appropriately processed salt-processed *Semen Cuscutae* sample.

#### 2.2.5. Preparation of the Over-Processed Sample

Stir-fry for 7 min until the surface of the herbal pieces turns dark brown, cracks, and emits a scorched aroma. Quickly remove from the wok, spread evenly to cool, and obtain the over-processed salt-processed *Semen Cuscutae* sample.

#### 2.2.6. Sample Preparation

The salt-processed *Semen Cuscutae* pieces from the four processing degrees—S, BG, SZ, and TG—were pulverized and passed through a No. 4 sieve to obtain the test samples. All sample powders were stored in a dry, sealed, and light-protected environment.

#### 2.2.7. Determination of Moisture Content in Raw and Processed Samples

Raw *Semen Cuscutae* and salt-processed *Semen Cuscutae* samples with different processing degrees were rapidly crushed and sieved through a No. 2 sieve. Accurately weigh 2–3 g of sample and spread it evenly in a flat weighing bottle that has been pre-dried to constant weight, with the sample layer thickness no more than 5 mm. The weighing bottle cap was opened, and the bottle was placed in an oven and dried at 100–105 °C for 5 h. The cap was then closed; the bottle was transferred into a desiccator and cooled for 30 min before accurate weighing. Drying for another 1 h under the same conditions, followed by cooling and weighing, was repeated until the mass difference between two consecutive weighments was ≤5 mg, which was regarded as constant weight. The moisture content (%) was calculated according to the mass loss of the sample before and after drying. Four groups of raw and processed samples were set in this study, with 15 biological replicates in each group. Each biological replicate was subjected to 2 technical replicate measurements. The average value of the two technical replicates was calculated for each individual sample, and the moisture content of each group was calculated based on the mean values of the 15 biological replicates. The formula for moisture content (%) is as follows:

[(m_1_ − m_2_)/(m_1_ − m_0_)] × 100%

m_0_ = mass of empty weighing bottle (g); m_1_ = mass of weighing bottle plus sample before drying (g); m_2_ = mass of weighing bottle plus sample after drying to constant weight (g).

### 2.3. Machine Vision

The image acquisition of *Semen Cuscutae* samples was carried out entirely in an intelligent DEEP Depu small photography studio (60 cm × 60 cm × 60 cm). LED soft light sources were symmetrically arranged on both sides and the top of the studio. The angle between the light sources and the shooting table was fixed at 45°, and the height of the light sources above the sample shooting plane was uniformly 35 cm. LED light sources with a standard natural light color temperature of 5500 K and a color rendering index (CRI) ≥ 92 were used, with stepless constant brightness output and no brightness fluctuation throughout the shooting process. The camera was vertically fixed directly above the sample, and the lens-to-sample-table distance was uniformly 50 cm. The mechanical position was fixed throughout the process, with no manual handheld deviation error. A white PVC background board was uniformly used for shooting. A camera was used as the tool, and before each acquisition, an A4 paper was used to calibrate the camera white balance to ensure the standard and reproducibility of image color. This study included four processing groups, with 15 independent stir-frying biological batches per group, totaling 60 independent sample batches. Each batch was randomly spread and evenly placed, with uniform sample orientation and spreading thickness, without stacking or occlusion. Four images were collected separately for each batch, totaling 240 images. Images of *Semen Cuscutae* with varying degrees of processing were captured using a Sony A7C2. The specific shooting parameters were as follows: White Balance: 6500 K; Aperture: F10; Shutter Speed: 1/200 s; ISO: 125. The images were saved in JPG format. The original images had pixel dimensions of 7008 × 4672 pixels (approximately 32.8 MP) and a resolution of 300 dpi. Images were named according to the pattern “treatment_group_batch_replicate” (e.g., S-1-1 jpg).

#### Extracting RGB Color Values

In the subsequent analysis, 30 images were randomly selected from each group, totaling 120 images for color feature extraction. In the RGB color space, the spatial coordinate axes are the three primary colors: red (R), green (G), and blue (B). Adobe Photoshop 2024 was used to separate the main body of the decoction piece image from the background. The background was filled with black, and the main body of the decoction pieces was defined as the region of interest (ROI). PyCharm Community Edition 2023.2 (JetBrains s.r.o., Prague, Czech Republic) software was used to quantify the pixels within the ROI. The sums of the R, G, and B values of all pixels within the ROI and the total number of pixels within the ROI (N_ROI_) were calculated. Black background pixels were excluded from the statistics. The average R, G, and B values of the decoction piece body were calculated using Equations (1)–(3) to perform calculations.

(1) $$R=\frac{{\mathrm{Sum}\;\mathrm{value}\;\mathrm{of}\;R}_{\mathrm{ROI}}}{N_{\mathrm{ROI}}}$$

(2) $$G=\frac{{\mathrm{Sum}\;\mathrm{value}\;\mathrm{of}\;G}_{\mathrm{ROI}}}{N_{\mathrm{ROI}}}$$

(3) $$B=\frac{{\mathrm{Sum}\;\mathrm{value}\;\mathrm{of}\;B}_{\mathrm{ROI}}}{N_{\mathrm{ROI}}}$$

### 2.4. Electronic Nose Analysis

Four samples of salt-processed *Semen Cuscutae* with different degrees of processing were pulverized and all passed through a No. 4 sieve. Then, 2.0 g of salt-processed *Semen Cuscutae* powder from each independent stir-frying batch was accurately weighed, placed in a 50 mL beaker, sealed with sealing film, and incubated statically for 24 h at room temperature (25 ± 1 °C) in the dark before detection. Headspace sampling was used to collect sample odor signals; the electronic nose sampling needle was inserted into the sealed beaker for data acquisition. The injection gas flow rate was set at 200 mL/min; the sensor self-cleaning time was 90 s; the sample preparation pre-equilibration time was 5 s; and the signal analysis acquisition time was 120 s. The response index (e.g., G/G_0_) was defined as the ratio of the real-time signal value to the initial baseline signal. During the 120 s continuous signal acquisition, the steady-state interval from 60 s to 120 s was selected to extract quantitative data for statistical analysis, avoiding the unstable fluctuation phase at the initial stage of injection. Before each sample measurement, sensor baseline calibration was performed using clean, dry air, and the detection environment was maintained at 25 ± 1 °C throughout. In this study, each group included 15 independent stir-frying biological batches (*n* = 15 per group, with four groups in total), the total dataset contained 60 independent batches across four groups, and each independent sample underwent five technical replicate acquisitions for subsequent odor characteristic analysis. Statistical analysis was performed using WinMuster software (1.6.2, G.A.S. mbH, Dortmund, Germany) [11].

### 2.5. Electronic Tongue Analysis

The electronic tongue system employed taste sensors, with Ag/AgCl as the reference electrode. Measurements were performed at room temperature (25 ± 1 °C) to detect the following sample attributes: saltiness, sourness, umami, bitterness, astringency, aftertaste-A, aftertaste-B, and richness, and to evaluate the overall taste profile. Before detection, the sensors were preheated and conditioned for 30 min and calibrated using the standard reference solution provided by Insent. Baseline calibration was performed before daily detection and before and after each batch of sample measurement to ensure baseline drift < 5 mV. After each sample measurement, the electronic tongue-specific cleaning solution was used for 90 s, repeated twice, until the baseline recovered. A blank solution was inserted to confirm no sample carryover. If the residual signal exceeded 5%, additional cleaning cycles were added. The sample measurement order was randomized to reduce systematic error. After the sensors were activated and calibrated, the samples were measured. The data acquisition time was set to 150 s, with a sampling interval of 1.00 s. For each type of salt-processed *Semen Cuscutae* powder with different processing degrees (passed through a No. 4 sieve), 1.0 g was accurately weighed. Then, 100 mL of water was added, stirred, and then it was allowed to stand for 30 min. Subsequently, the mixture was subjected to ultrasonic treatment for 1 h, followed by cooling and standing. The supernatant was collected, filtered, and the filtrate was transferred into a dedicated 50 mL electronic tongue beaker for computer-based detection. Each group included 15 independent parallel samples, and each sample was continuously measured for 4 cycles. After excluding unqualified cycles, the arithmetic mean of the qualified cycles was used as the sole representative value of the sample for statistical analysis (*n* = 15 per group), and the total dataset contained 60 independent batches across four groups [12].

### 2.6. High-Performance Liquid Chromatography Analysis

#### 2.6.1. Preparation of Reference Standard Solutions

Appropriate amounts of hyperoside, astragalin, isoquercitrin, quercetin, and kaempferol reference standards were accurately weighed, and 80% methanol was added to prepare a mixed reference standard solution containing 506.00 μg hyperoside, 202.80 μg astragalin, 203.00 μg isoquercitrin, 18.20 μg quercetin, and 20.06 μg kaempferol per 1 mL.

#### 2.6.2. Preparation of Test Sample Solutions

Salt *Semen Cuscutae* powder with different processing degrees (passed through a No. 4 sieve) was precisely weighed (1 g) and placed in a 50 mL volumetric flask. Then, 40 mL of 80% methanol was added. Subsequently, the mixture was subjected to ultrasonic treatment (power: 100 W, frequency: 40 kHz) for 15 min. The temperature of the ultrasonic water bath was set at 30 °C. After cooling, the volume was made up to the mark with 80% methanol, and the mixture was shaken well. Finally, it was filtered through a 0.45 μm organic membrane filter, and the successive filtrate was collected as the test sample solution.

#### 2.6.3. Chromatographic Conditions

The separation was performed on a YMC-Pack ODS-A column (250 mm × 4.6 mm, 5 μm). The mobile phase consisted of acetonitrile (A) and aqueous phosphoric acid solution (B). Gradient elution was carried out as follows: 0–30 min, 12–22% A; 30–50 min, 22–26% A; 50–65 min, 26–40% A; 65–70 min, 40–12% A. The column temperature was 35 °C, the flow rate was 1.0 mL/min, the detection wavelength was 360 nm. The HPLC injection volume was 10 μL, and the total chromatographic run time was 70 min. Each group contained 15 independent parallel samples, and each sample was measured continuously 3 times [13].

#### 2.6.4. Linearity and Method Validation

Linearity: An appropriate amount of the above mixed reference standard solution was taken and sequentially diluted 2-, 5-, 10-, 15-, and 25-fold to obtain reference standard solutions at each gradient. Then, 10 μL of each gradient reference standard solution was injected. Using the reference standard concentration (μg·mL^−1^) as the abscissa X and the peak area as the ordinate Y, the regression equation and regression coefficient were calculated.

Precision: A total of 200 μL of the mixed reference standard solution was accurately pipetted into an injection vial and injected continuously 6 times under the chromatographic conditions. The peak areas were measured, and the relative standard deviation (RSD) was calculated.

Stability test: Four samples of salt-processed *Semen Cuscutae* with different processing degrees were taken, and one test solution (200 μL) was prepared for each sample. At 0, 2, 4, 8, 12, and 24 h, each test solution was accurately pipetted and injected for determination under the chromatographic conditions. The peak areas were recorded, and the RSD was calculated.

Repeatability: Four types of salt-processed *Semen Cuscutae* samples with different degrees of processing were taken. For each sample, six test solutions were prepared in parallel, processed according to the test solution preparation method, and detected under the chromatographic conditions. The content of each component and the RSD were calculated.

Spiked recovery: Four types of salt-processed *Semen Cuscutae* samples with different degrees of processing were taken, and six test solutions were prepared for each type. The solutions were placed in conical flasks, and appropriate amounts of stock solutions of hyperoside, isoquercitrin, astragalin, quercetin, and kaempferol reference standards were accurately added. After sample pretreatment, the solutions were determined under the chromatographic conditions, and the spiked recovery was calculated.

### 2.7. GC-IMS Analysis

A FlavourSpec^®^ GC-IMS system equipped with an MXT-WAX capillary column (15 m × 0.53 mm, 1.0 μm film thickness; Restek, Bellefonte, PA, USA, with a polyethylene glycol (PEG) stationary phase) was used and a CTC-PAL automatic sample injection system. Both the carrier gas and drift gas were high-purity nitrogen (≥99.999%). VOCal data processing software (0.4.10: G.A.S. Germany).

#### 2.7.1. Headspace Sampling and Sample Preparation

Different processing degrees of salt-processed *Semen Cuscutae* powder (passed through a No. 4 sieve (250 ± 9.9 μm)) were accurately weighed (0.2 g), and 10 μL of 100 mg/L 2-octanol solution was added as the internal standard. After incubation at 80 °C for 15 min, proceed with injection. 2-Octanol was used to monitor injection repeatability and IMS response stability, and for normalization correction of target peak intensities. For each independent stir-frying batch, three technical headspace injection replicates were performed, and the arithmetic mean of the three technical replicates was used as the sole representative value for that batch for subsequent statistical analysis *(n* = 15 per group), and the total dataset contained 60 independent batches across four groups. The three technical replicates were only used to evaluate injection repeatability and were not regarded as independent replicates. A headspace sampling method was employed with an injection volume of 100 µL per sample, splitless mode, and syringe temperature of 110 °C. Using the peak volume of 2-octanol as the internal standard, the peak volume of each target compound was divided by the internal standard peak volume to obtain the relative peak volume. This relative peak volume was used for semi-quantitative comparison among groups. For each independent stir-frying batch, three technical headspace injection replicates were performed, and the arithmetic mean of the three technical replicates was used as the sole representative value for that batch. Statistical analysis was performed based on 15 independent stir-frying batches per group, and data are expressed as mean ± SD (*n* = 15 per group, four groups in total). The RSD of technical replicates was used as an indicator of injection repeatability and uncertainty. Absolute quantification was not performed in this study, and all GC-IMS data only represent relative response changes and cannot be directly equated with the total concentration of compounds. The built-in GC retention index database (NIST 2020) and IMS drift time database in VOCal software were used for retrieval and comparison. The allowed deviation for retention index matching was set at ±20; the relative deviation for drift time matching was ≤3%; the database matching similarity threshold was ≥70%. Only when both the retention index and drift time met the above thresholds were the compounds included in tentative identification.

#### 2.7.2. GC Conditions

Column Temperature Control: The column was kept at a constant 60 °C. Gradient Flow Rate Program: Initial Stage (0–2 min): Constant flow rate of 2.0 mL/min. First Gradient Stage (2–10 min): Linearly increased to 10.0 mL/min over 8 min. Second Gradient Stage (10–20 min): Further linearly increased to 100.0 mL/min over the next 10 min. Steady-State Stage (20–50 min): Maintained at the final flow rate of 100.0 mL/min for 30 min. Injection Parameters: The injector temperature was held constant at 80 °C. Timing Setup: The total analysis cycle was 50 min.

#### 2.7.3. IMS Conditions

Ionization source: Tritium source (^3^H). Migration tube length: 53 mm. Electric field strength: 500 V/cm. Positive ion mode, gate opening time 100 μs, gate closing voltage 90 dgt. Migration tube temperature: 45 °C. Flow rate: 75.0 mL/min [9].

### 2.8. Antioxidant Analysis

Accurately weigh 1.0 g of salt-processed *Semen Cuscutae* powder (passed through No. 4 pharmacopeia sieve). Add 15 mL of 90% ethanol solution at a solid–liquid ratio of 1:15 (g/mL), and perform a heating reflux extraction at 75 °C for 100 min. The extract was centrifuged at 8000 r/min for 10 min at room temperature. The supernatant was collected and diluted to 15 mL to obtain the sample stock solution. The stock solution was used directly as the test solution without further dilution (dilution factor = 1) for subsequent DPPH, ABTS and FRAP antioxidant assays.

DPPH radical scavenging assay. The assay was performed using a DPPH antioxidant detection kit (microplate method, 96 samples) from Xiamen Lanchangshuo Biotechnology Co., Ltd., Xiamen, Fujian, China. The DPPH working solution and a series of Trolox standard solutions were prepared according to the kit instructions. The reaction system was prepared in EP tubes as follows: test tube: 150 μL sample test solution + 150 μL DPPH working solution; control tube: 150 μL 80% methanol + 150 μL sample test solution; blank tube: 150 μL 80% methanol + 150 μL working solution. After thorough mixing, the mixture was kept in the dark at 25 °C for 30 min. The mixture was then centrifuged at 12,000 r/min for 5 min, and 200 μL of supernatant was transferred to a 96-well microplate. The absorbance was measured at 517 nm using an EnSight multimode microplate reader. The calibration curve was plotted with Trolox concentration (μg/mL) as the abscissa and scavenging rate (%) as the ordinate: y = 3.04x − 0.6667, R^2^ = 0.999. The antioxidant activity of samples was converted to Trolox equivalent, expressed as μg TE/g dry weight (DW). All assays were carried out in triplicate. DPPH radical scavenging rate (%) = [1 − (A sample − A control)/A blank] × 100%. A sample: Absorbance of sample test solution mixed with DPPH working solution. A control: Absorbance of 80% methanol mixed with sample test solution. A blank: Absorbance of 80% methanol mixed with working solution.

The ABTS radical scavenging capacity was determined according to the instructions of the Solarbio BC4770 assay kit. Trolox was used as the standard, and Trolox standard solutions were prepared with the extraction solution. The ABTS working solution was freshly prepared by mixing Reagent 1, Reagent 2 and working solution of Reagent 3 at a volume ratio of 76:5:4, and used within 30 min at room temperature under dark conditions. Reagents were added sequentially to EP tubes: 50 μL of distilled water, 100 μL of working solution of Reagent 4 and 850 μL of ABTS working solution were added to the blank tube; 50 μL of sample supernatant, 100 μL of working solution of Reagent 4 and 850 μL of ABTS working solution were added to the sample determination tube; 50 μL of sample supernatant, 850 μL of ABTS working solution and 950 μL of Reagent 1 were added to the sample control tube. After reagent addition, the mixtures were fully mixed and incubated at room temperature in the dark for 6 min. The absorbance of each tube was measured at 734 nm and recorded as A blank, A sample and A control, respectively. The standard curve was plotted with Trolox concentration (μg/mL) as the abscissa and scavenging rate (%) as the ordinate: y = 3.0457x − 0.5714, R^2^ = 0.9992. The antioxidant activity of samples was converted into Trolox equivalents according to the standard curve, expressed as μg TE/g dry weight (DW). Three replicates were set for each group. ABTS radical scavenging rate (%) = [1 − (A sample − A control)/A blank] × 100%. A blank is the absorbance of the blank tube; a sample is the absorbance of the reaction system containing sample and ABTS; a control is the absorbance of the sample background control.

FRAP ferric reducing antioxidant power assay. FRAP assay was carried out using a FRAP antioxidant kit from Yuanye Bio-Technology. Fresh FRAP working solution was prepared according to the kit instruction (FRAP Assay Buffer:TPTZ solution:ferric chloride solution = 10:1:1, prepared freshly and used within 2 h). A series of Fe^2+^ standard solutions were prepared. The reaction system was prepared in EP tubes: test tube: 180 μL sample test solution + 1584 μL FRAP working solution; standard tube: 180 μL gradient Fe^2+^ standard solution + 1584 μL FRAP working solution; blank tube: 180 μL distilled water + 1584 μL FRAP working solution. After mixing, the mixture was incubated in a water bath at 37 °C in the dark for 30 min. The supernatant was transferred to a 96-well microplate, and absorbance was measured at 593 nm using the EnSight multimode microplate reader. The calibration curve was plotted with Fe^2+^ concentration (mmol/L) as the abscissa and absorbance as the ordinate: y = 1.7701x + 0.0944, R^2^ = 0.9992. The Fe^2+^ equivalent concentration (mmol/L) of samples was calculated based on the calibration curve. All assays were performed in triplicate.

### 2.9. AHP-TOPSIS Analysis

#### 2.9.1. Analytic Hierarchy Process

Based on the subjective evaluation combined with the importance of each evaluation indicator for salt-processed *Semen Cuscutae* with different processing degrees, the content of hyperoside, the content of astragalin, appearance characters, W1W, W2W, richness, and the total peak volume intensity values of differential volatile components (VIP > 1) in flavor substances were used as weight indicators and were quantified. The evaluation criteria for the appearance character of salt-processed *Semen Cuscutae* and the comprehensive scores of appearance character for salt-processed *Semen Cuscutae* with different processing degrees are presented in Table S3 and Table S4, respectively. A pairwise comparison judgment matrix was constructed for the aforementioned seven indicators, and the results are presented in Table S5 [14].

#### 2.9.2. Entropy Weight Method

The entropy weight method determines weights based on the data itself and completes the evaluation through objective weighting. It provides an objective approach for weight determination. The original data of m indicators across n experimental groups were subjected to forward normalization, non-negative translation, and normalization processing. The processed data is analyzed by entropy weight, and the ω_EW,j_ was obtained.

#### 2.9.3. AHP-Entropy Weight Combined Weighting Method

The subjective weights derived from AHP and the objective weights obtained by the entropy weight method were coupled to generate combined weights, which could reflect sample information more comprehensively and objectively. The formula for calculating the weight coefficient is $\omega_{c,j}=\frac{\omega_{\mathrm{AHP},j}\cdot \omega_{\mathrm{EW},j}}{\sum_{j=1}^{n}(\omega_{\mathrm{AHP},j}\cdot \omega_{\mathrm{EW},j})}$. The subscript j denotes the serial number of evaluation indicators. The ω_AHP,j_ is the weight coefficient obtained by AHP method and ω_EW,j_ is the weight coefficient obtained by entropy weight method [15].

#### 2.9.4. Calculation of the AHP-TOPSIS Model

First, the analytic hierarchy process (AHP) was used to obtain the subjective weights of indicators, and the entropy weight method was adopted to calculate the objective weights of indicators. The combined weights were obtained by coupling the subjective and objective weights. After weighting the standardized decision matrix with the combined weights, the positive ideal solution S^+^ and negative ideal solution S^−^ were determined. The distances from each sample to the positive ideal solution $D_{i}^{+}$ and the negative ideal solution $D_{i}^{-}$ were calculated, respectively. The relative closeness C_i_ was calculated according to the following formula: [16].

$$\left(C_{i}=\frac{D_{i}^{-}}{D_{i}^{+}+D_{i}^{-}}\right)$$

The samples were ranked by the value of Ci. A higher Ci value indicates better comprehensive performance of the sample.

### 2.10. Data Analysis

All statistical analyses and plotting were performed using Origin 2024 (OriginLab, Northampton, MA, USA), SIMCA 14.1 (Umetrics, Umea, Sweden), The Unscrambler X 9.7 (CAMO, Oslo, Norway), and the Metware Cloud Platform (Metware Biotechnology, China).

The experimental unit was an independent batch of stir-fried, salt-prepared *Semen Cuscutae*. Four processing degrees (S, BG, SZ, TG) were investigated in this study, with 15 independent batches per group (*n* = 15 per group; total N = 60). For technical replicates including five acquisition cycles of electronic nose detection, four acquisition cycles of electronic tongue detection, and three headspace injections of GC-IMS, the average value within each independent batch was calculated first to obtain a single representative value for that batch. Technical replicates were not treated as independent samples. Data were expressed as mean ± SD. The Shapiro–Wilk test was used to assess normality, and the Levene test for homogeneity of variances. One-way analysis of variance (one-way ANOVA) followed by Tukey’s HSD post hoc test was adopted for intergroup comparisons when the assumptions were satisfied. If the assumptions were violated, the Kruskal–Wallis test with Dunn’s post hoc test and Bonferroni correction was applied. The significance level was set at α = 0.05. Effect sizes were reported as η^2^ for ANOVA or r for nonparametric tests, and Cohen’s d for pairwise comparisons where applicable. Multiple comparisons were controlled using Tukey’s HSD or Bonferroni correction. Data transformation (e.g., log10 or square-root transformation) was performed when necessary to meet normality or homoscedasticity assumptions, and the applied transformation was stated in figure legends. No missing values were present in this dataset; samples would be excluded if missing values occurred.

Prior to PCA, OPLS-DA, LDA and PLSR, variables were mean-centered and scaled to unit variance (UV scaling). PCA was conducted on batch-level mean values. The number of principal components was selected based on eigenvalues > 1 and scree plots, with cumulative explained variance reported. PCA was used as an unsupervised exploratory approach. OPLS-DA was implemented in SIMCA. The number of latent variables was determined via 7-fold cross-validation to minimize the root mean square error of cross-validation (RMSECV) and maximize Q^2^. Model validity was evaluated using R^2^Y, Q^2^ and a permutation test with 200 random permutations. The Q^2^ intercept obtained from permutation testing served as an indicator for overfitting; a negative Q^2^ intercept suggested no model overfitting. The permutation test was not interpreted as a *p*-value. To avoid data leakage, technical replicates from the same independent batch were always kept in the same training or test fold; cross-validation was performed at the batch level.

LDA was implemented using Origin 2024 and SIMCA. Model performance was evaluated by batch-level leave-one-out cross-validation (LOOCV) or 7-fold cross-validation. Confusion matrices and overall accuracy were reported wherever classification outcomes were claimed. PLSR was constructed in The Unscrambler X 9.7 to analyze linear relationships between independent variables (e.g., sensor responses or component contents) and dependent variables. The optimal number of latent variables was determined by 7-fold cross-validation based on the minimum root mean square error of prediction (RMSEP). Model performance was reported in terms of R^2^, Q^2^ and RMSEP. Hierarchical clustering heatmaps were generated on the Metware Cloud Platform using Euclidean distance and Ward’s linkage method. Pearson correlation coefficients were used to construct correlation heatmaps. Multiple testing correction was applied where appropriate. Given the limited number of independent processing batches, supervised models (OPLS-DA, LDA and PLSR) were mainly used for exploratory interpretation and pattern recognition rather than for robust predictive conclusions. Predictive performance should be interpreted cautiously, and external validation on a larger independent cohort is warranted.

## 3. Results

### 3.1. Machine Vision Analysis

**Figure 1:**
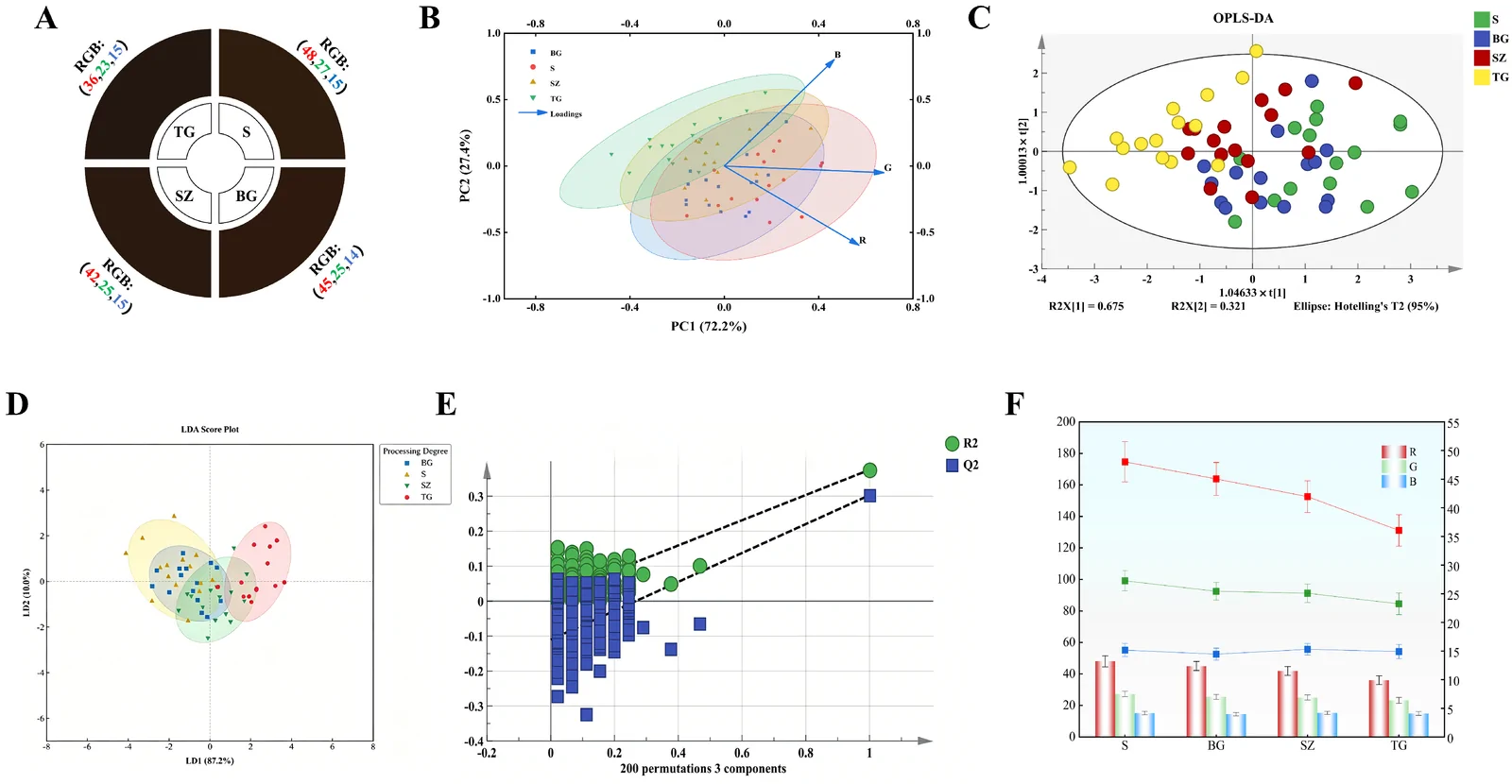
Salt-processed *Semen Cuscutae* with different processing degrees. (**A**) RGB color images. (**B**) PCA plot. (**C**) OPLS-DA plot. (**D**) LDA plot. (**E**) OPLS-DA permutation test plot. (**F**) Combined line and bar chart. (*n* = 15 per group, four groups in total. PCA, principal component analysis. OPLS-DA, orthogonal partial least-squares discriminant analysis. R, red channel; G, green channel; B, blue channel; LDA, Linear Discriminant Analysis. R^2^, goodness-of-fit parameter; Q^2^, predictive ability parameter).

Salt-processed *Semen Cuscutae* samples at different processing stages exhibited significant differences (Figure 1A).

Based on Python algorithms, the average RGB values of salt-processed *Semen Cuscutae* were extracted. The surface of the seeds exhibited colors ranging from grayish-brown to brownish-yellow. During the salt-processing, with the extension of stir-frying time (Figure 1F), the color gradually darkened, with the R and G values decreasing significantly while the B value remained relatively stable. This indicates that the color changes were concentrated in the red-green spectrum.

Principal component analysis (PCA) and orthogonal partial least squares discriminant analysis (OPLS-DA) models showed (Figure 1B,C) that the discrimination ability for salt-processed *Semen Cuscutae* with different processing degrees was weak. LDA showed a relatively good trend in class discrimination, with contribution rates of 87.2% for LD1 and 10.0% for LD2. However, its discriminative ability still needs to be further validated in combination with classification accuracy and cross-validation results.

To validate the robustness of the OPLS-DA model, 200 permutation tests were performed (Figure 1E). The results showed that the intercept of the Q^2^ regression line was −0.33, which was below the overfitting threshold used in this study (Q^2^ intercept < 0.05). Meanwhile, both the R^2^ and Q^2^ permutation regression lines exhibited downward trends with negative intercepts. According to this criterion, the model did not show overfitting and demonstrated good statistical reliability [17].

### 3.2. Electronic Nose Result Analysis

As shown by the 2D principal component analysis (PCA) combined with bar chart, the raw products, under-processed products, appropriately processed products, and excessively processed products were clearly separated on principal component 1 and principal component 2. Among them, the raw and under-processed samples have a relatively weak discrimination effect in terms of odor information, showing a tendency to cluster into one category. Overall, the sample points can be divided into three relatively independent regions, indicating that the odor characteristics of samples with different stir-frying degrees have significant differences. The appropriately processed samples are located in the middle position in the PCA plot, and their odor information dominates the overall distribution. The PCA results of this study show a clear separation of various samples, indicating that the processing treatment has caused significant changes in the internal metabolite composition of the samples. This change is consistent with the trend presented by the sample phenotypes (such as appearance indicators).

**Figure 2:**
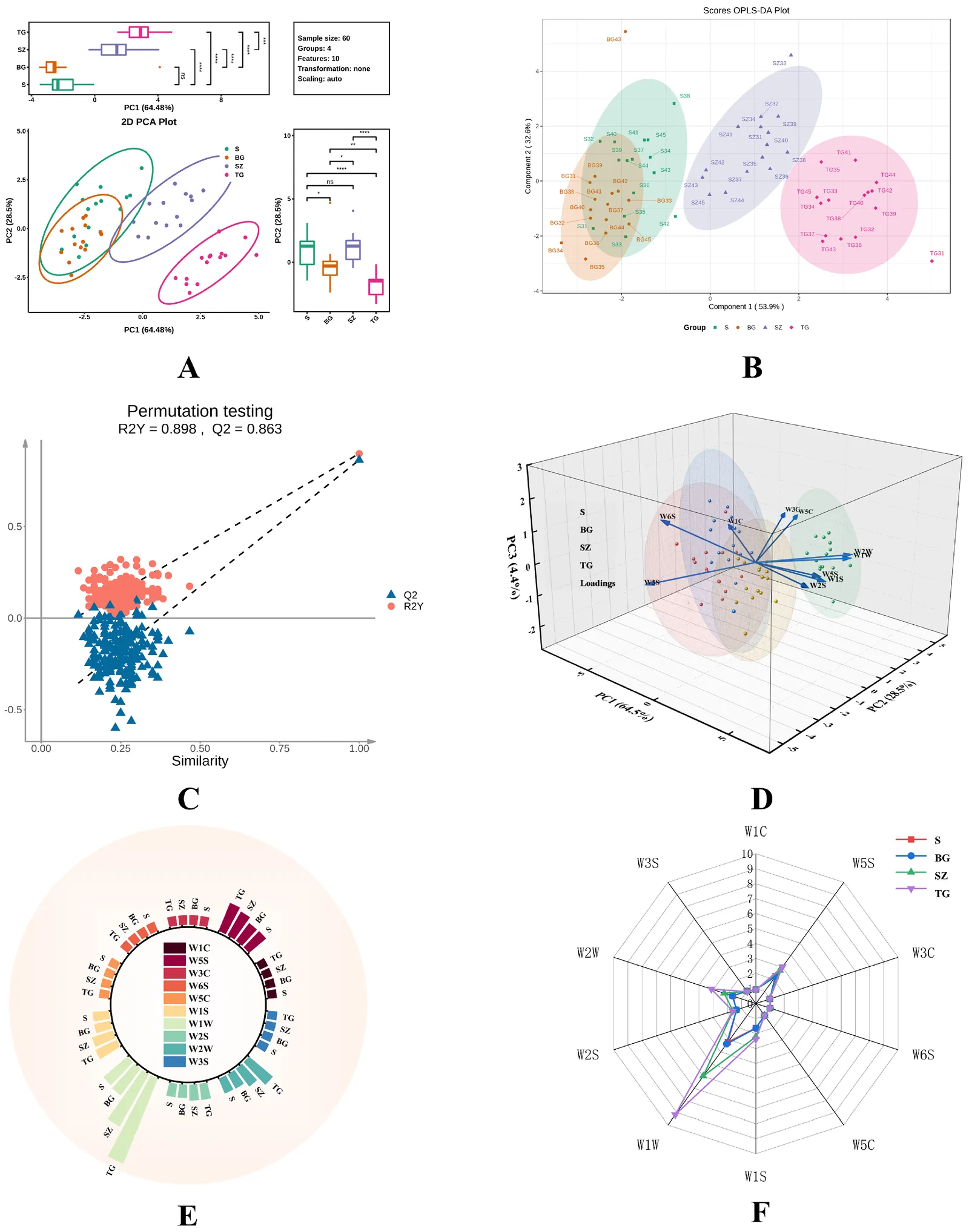
Odor profiles of salt-processed *Semen Cuscutae* with different processing degrees. (**A**) PCA combined bar chart. (**B**) OPLS-DA plot. (**C**) OPLS-DA permutation test plot. (**D**) 3D PCA plot. (**E**) Bar chart. (**F**) Electronic nose radar chart. (*n* = 15 per group, four groups in total. PCA, principal component analysis. OPLS-DA, orthogonal partial least-squares discriminant analysis. W1C, W5S, W3C, W6S, W5C, W1S, W1W, W2S, W2W, W3C are ten metal oxide sensors of PEN3 electronic nose, with their sensitive volatile substances described above. * *p* < 0.05, ** *p* < 0.01, *** *p* < 0.001, **** *p* < 0.0001, ns = not significant (*p* ≥ 0.05).

A further orthogonal partial least squares discriminant analysis (OPLS-DA) model was established based on the electronic nose, and the results are shown in Figure 2B. The raw products, under processed products, appropriate products and excessive products are also effectively distinguished in terms of composition. Among them, the raw products and the under processed products still show a clustering tendency, and the overall distribution is in three categories. The permutation test results of the OPLS-DA model (Figure 2C) show that R^2^ = 0.898 (>0.5) and Q^2^ = 0.863 (>0.5). It indicates that the established model has good stability and reliability, and is suitable for discriminant analysis of the processing degree of stir-fried *Semen Cuscutae*.

The electronic nose radar chart of different processing degrees of salt-processed *Semen Cuscutae* is shown in Figure 2F. The results indicate that as the degree of frying increases, the electronic nose signals of appropriately fried salt-processed *Semen Cuscutae* show significant differences from those of raw, under-processed and over-processed products [18].

### 3.3. Electronic Tongue Results Analysis

**Figure 3:**
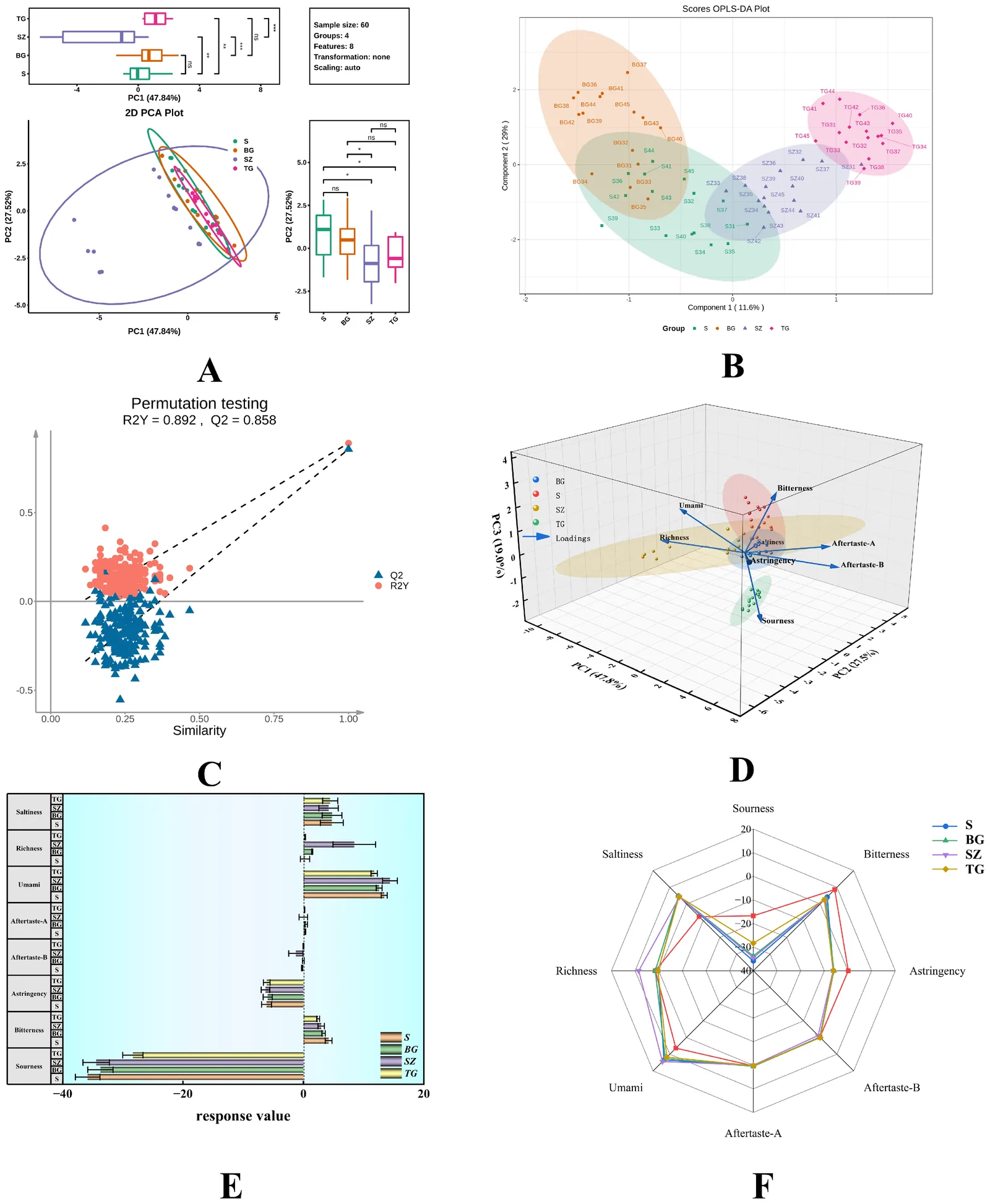
Taste profiles of salt-processed *Semen Cuscutae* with different processing degrees. (**A**) PCA combined bar chart. (**B**) OPLS-DA plot. (**C**) OPLS-DA permutation test plot. (**D**) 3D PCA plot. (**E**) Bar chart. (**F**) Electronic tongue radar chart. (*n* = 15 per group, four groups in total. PCA, principal component analysis. OPLS-DA, orthogonal partial least-squares discriminant analysis. CA0, C00, AE1, AAE, CT0, GL1, AN0, BT0 are lipid membrane sensors of the Insent SA402B electronic tongue for taste detection * *p* < 0.05, ** *p* < 0.01, *** *p* < 0.001, ns = not significant (*p* ≥ 0.05).

Taste response signals were collected for the aqueous extracts of salt-processed *Semen Cuscutae* samples with different processing degrees. The data were analyzed using a 2D principal component analysis (PCA) combined with a column chart. On this basis, orthogonal partial least squares discriminant analysis (OPLS-DA) was further employed to establish a classification discrimination model. The model’s stability and reliability were verified through permutation tests. Eventually, precise discrimination of salt-processed *Semen Cuscutae* samples with different processing degrees was achieved. As shown in Figure 3, the electronic tongue signals of differently processed salt-processed *Semen Cuscutae* prepared in this study exhibited distinct differentiation. In the orthogonal partial least squares-discriminant analysis (OPLS-DA), the over-processed samples showed significant separation from the raw products, under-processed, and appropriately processed samples in the reduced-dimensional space. Electronic tongue results showed that compared with the raw, under-processed, and appropriately processed samples, the over-processed (TG) samples had significantly higher response values related to umami and taste richness. Compared with the raw products, the appropriately processed group (SZ) showed reduced bitterness and astringency and an improved taste profile [19].

### 3.4. High-Performance Liquid Chromatography (HPLC) Analysis

#### 3.4.1. Linearity

The linear regression equations of the reference standards are shown in Table 1. The results showed that the peak areas of the five components had good correlation with concentration.

#### 3.4.2. Method Validation

The precision RSD, LOD and LOQ data of the five compounds are summarized in Table 2, while the stability, repeatability and spiked recovery RSD results are presented in Table 3. The precision RSDs of hyperoside, astragalin, isoquercitrin, quercetin, and kaempferol were <3%, indicating good instrument precision. The stability RSDs of these five components were all <3%, indicating that the test solution was stable within 24 h. The repeatability RSDs of the five components were all <3%, indicating good repeatability of the extraction method. The average spiked recoveries ranged from 93.21% to 102.62%, with RSD < 4%, indicating good recovery of the method.

#### 3.4.3. Analysis of HPLC Results

**Figure 4:**
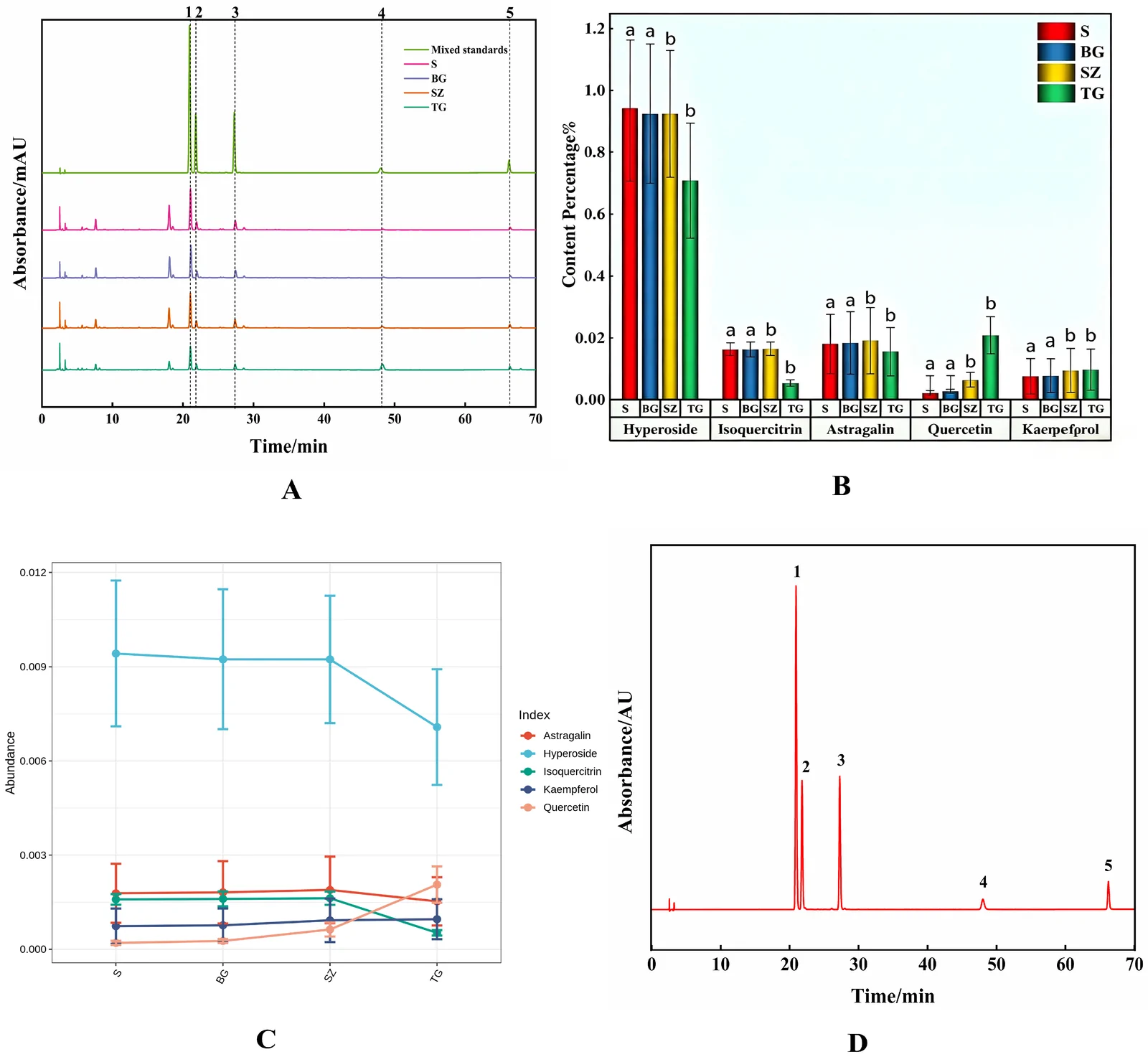
HPLC fingerprinting of salt-processed *Semen Cuscutae* with different processing degrees. (**A**) HPLC fingerprints. (**B**) Bar chart. (**C**) Line chart. (**D**) HPLC fingerprint. (Peak assignments: 1, hyperoside; 2, isoquercitrin; 3, astragalin; 4, quercetin; 5, kaempferol. n = 15 per group, four groups in total. One-way ANOVA followed by Tukey’s HSD test, *p* < 0.05. Different lowercase letters indicate significant differences at *p* < 0.05; the same letter denotes no significant difference (*p* ≥ 0.05). The contents of all flavonoid components are expressed in %).

HPLC fingerprint of five flavonoid components in salt-processed *Semen Cuscutae* samples with different processing degrees revealed that the processing procedure significantly affected their chemical composition. Figure 4 illustrates the chromatographic response characteristics of hyperoside, isoquercitrin, astragalin, quercetin, and kaempferol in mixed standards and processed samples (S, BG, SZ, TG). As shown in Figure 4A, all components were well-separated under the chosen chromatographic conditions. The mixed standard chromatogram displayed symmetrical peak shapes for each component, indicating good specificity of the analytical method. The chromatograms of samples with different processing degrees show significant variations in the response values of characteristic peaks with changes in the processing conditions. It is speculated that flavonoid components may undergo transformation and degradation during processing [20].

As shown in Figure 4B: Hyperoside exhibited the highest content among all samples. No significant difference was observed in its content between raw products (S), under-processed (BG), and appropriately processed (SZ) samples. However, its content significantly decreased in over-processed (TG) samples, suggesting that prolonged processing time may lead to its degradation. Isoquercitrin and astragalin: The content levels of these two components were similar across differently processed samples. However, the Isoquercitrin content in TG samples was significantly lower than in other samples, further confirming the impact of processing time on flavonoid glycosides. Quercetin and kaempferol contents exhibited a gradually increasing trend with increasing processing degree, and the TG group was significantly higher than the S, BG, and SZ groups.

As observed in Figure 4C, distinct differences exist in the “characteristic profiles” of the processed samples. The compositional proportion structure varies between the raw product (S) and the processed samples (BG, SZ, TG). Hyperoside remained the relatively dominant component in all samples, while quercetin and kaempferol consistently accounted for relatively low proportions [21].

In summary, the processing of salt-processed *Semen Cuscutae*, particularly processing duration, primarily influenced the contents of the dominant components, hyperoside and isoquercitrin, while the levels of aglycone components remained relatively stable.

### 3.5. GC-IMS Results Analysis

#### 3.5.1. Comparative Differential Analysis of Volatile Components in Salt-Processed *Semen Cuscutae* at Different Processing Degrees

**Figure 5:**
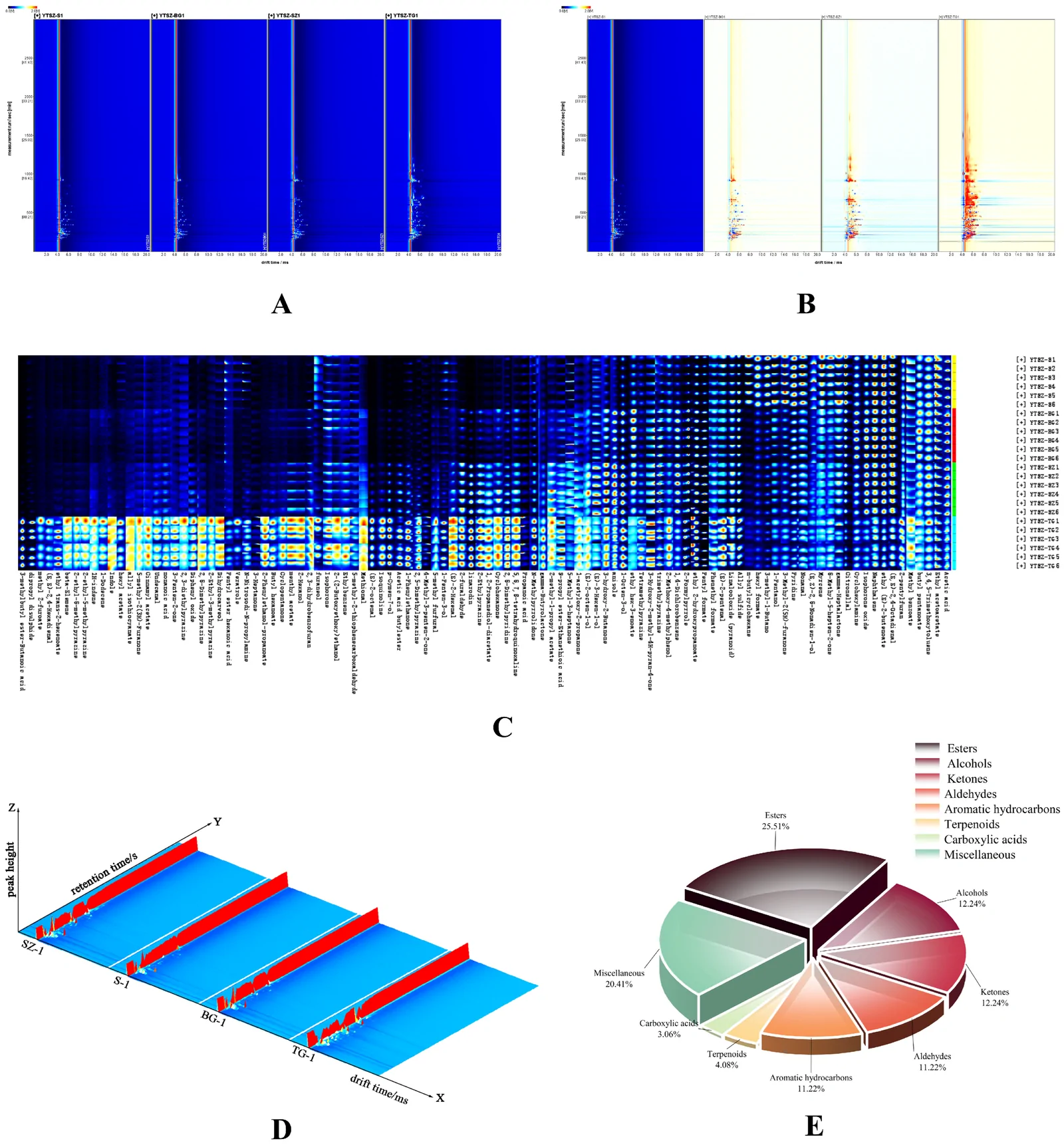
Volatile components analysis of salt-processed *Semen Cuscutae* with different processing degrees by GC-IMS. (**A**) GC-IMS 2D top view. (**B**) 2D differential spectrum. (**C**) Fingerprint. (**D**) GC-IMS 3D spectrum. (**E**) Classification and proportion chart of volatile components. (*n* = 15 per group, four groups in total. GC-IMS, gas chromatography–ion mobility spectrometry; VOCs, volatile organic compounds; Rt, retention time; Dt, drift time).

The VOCal software was used to perform differential analysis on the GC-IMS detection results, and three-dimensional spectra of volatile organic compounds (VOCs) were obtained and generated. All GC-IMS results are expressed as “semi-quantitative normalized results and it is clearly stated that they cannot replace absolute quantification. In these 3D spectra, each characteristic point corresponds to a volatile organic compound, and its color intensity visually represents the relative abundance of the detection signal for that component. As shown in Figure 5D, the three-dimensional axes of the spectrum represent: the X-axis is the ion migration time (ms), the Y-axis is the gas chromatographic retention time (s), and the Z-axis is the ion peak intensity. The analysis results indicate that the 3D spectrum of the over-processed, salt-processed *Semen Cuscutae* exhibits the highest quantity of ion peaks, suggesting the greatest variety of volatile flavor compounds. In contrast, the spectrum of the appropriately processed sample displays the highest ion peak signal intensity, indicating a relatively higher total content of volatile flavor substances. Notably, the volatile organic compounds in salt-processed *Semen Cuscutae* of different processing degrees exhibited significant differences in ion migration time. To enhance the visual discrimination of differences in volatile organic compounds (VOCs), dimensionality reduction was performed on the original data in this study [22].

To further intuitively reveal the differences among volatile flavor substances, two-dimensional profile spectra of salt-processed *Semen Cuscutae* at different processing degrees were plotted. As shown in Figure 5A, the red vertical line at the 1.0 mark on the x-axis is designated as the Reaction Ion Peak (RIP). Each independent point around the RIP peak corresponds to a volatile organic compound peak. Darker shades (approaching red) signify higher peak intensity, while lighter shades (approaching white) indicate lower peak intensity. By comparing the GC-IMS two-dimensional spectra of different samples, differences in the peak intensities of volatile organic compounds can be visually observed. In the spectrum of raw *Semen Cuscutae*, the feature points appear lighter in color, fewer in quantity, and smaller in shape, indicating a relatively lower number and content of volatile flavor compounds. By performing subtraction processing between the spectra of salt-processed *Semen Cuscutae* with different processing degrees (BG group, SZ group, TG group) and the reference spectrum (S group), differential spectra were obtained. In Figure 5B, the results demonstrated that gas chromatography–ion mobility spectrometry effectively separated the volatile compound profiles of salt-processed *Semen Cuscutae* with different processing degrees. Among the various samples, the content of some volatile compounds showed varying increases or decreases. The results showed notable differences between the SZ group and S group as well as the BG group, whereas the differences between the S group and the BG group were relatively minor. In the differential spectra of excessively processed salt-processed *Semen Cuscutae* (TG group), the number of red feature spots was greater than that of blue feature spots, indicating that the concentration of volatile components was significantly higher than that in the other three groups of samples. The results indicate that the degree of processing significantly affects the types and contents of volatile flavor compounds in salt-processed *Semen Cuscutae*.

#### 3.5.2. Qualitative Identification of Volatile Organic Compounds in Salt-Processed *Semen Cuscutae* with Different Processing Degrees

The VOCal data processing software was used for qualitative analysis of volatile compounds. 104 volatile compounds were tentatively identified (in Table S7). Among the tentatively identified compounds, esters accounted for 25.51%, alcohols and ketones each accounted for 12.24%, aldehydes and aromatic hydrocarbons each accounted for 11.22%, terpenes accounted for 4.08%, carboxylic acids accounted for 3.06%, and other categories accounted for 20.41% (Figure 5E). The volatile components of salt-processed *Semen Cuscutae* with varying degrees of processing are primarily composed of esters, alcohols, ketones, aldehydes, and aromatic hydrocarbons. Esters typically exhibit floral and fruity characteristics, while aldehydes and ketones often present herbal, medicinal, and floral notes [23].

#### 3.5.3. Comparative Analysis of Volatile Component Fingerprints in Salt-Processed *Semen Cuscutae* at Different Processing Stages

To systematically elucidate the quantitative differences in volatile components of salt-processed *Semen Cuscutae* at different processing degrees, this study employed the Gallery Plot plugin of the VOCal (0.4.10: G.A.S. Germany) software to construct volatile component fingerprints for the crude drug and salt-processed *Semen Cuscutae* with varying degrees of processing (under-processed, appropriately processed, and over-processed) (Figure 5C). All GC-IMS results in this study were analyzed and presented using a semi-quantitative normalization approach. Such semi-quantitative results have certain limitations and cannot replace precise absolute quantification of individual components. The fingerprints use a color gradient to represent the relative abundance of compounds, with the intensity of the red color being positively correlated with component content. The analysis revealed that while samples from different processing degrees share common volatile signals, they also exhibit significant characteristic differences in their spectral profiles. By comparing the intensities of key chromatographic peaks, it was found that the relative contents of some volatile organic compounds exhibited a significant dependence on the degree of processing. The specific manifestations are as follows: The raw *Semen Cuscutae* showed characteristically high signal responses for compounds such as citronellal, γ-heptalactone and myrcene. The under-processed products were characterized by significant peak intensities of substances such as ethyl 3-enoate and cyclohexylamine. The appropriately processed product exhibited dominant signals for anisole, 3-hydroxy-2-butanone, (E)-3-hexen-1-ol, and specific isomers of octadienal/butenoate ethyl esters. In contrast, the excessively processed products were characterized by significant enrichment of compounds such as (E)-2-octenal and 2-methoxy-4-methylphenol. These characteristic volatile components can serve as specific chemical markers for discriminating the volatile profiles of salt-processed *Semen Cuscutae* at different processing stages [24].

#### 3.5.4. Differential Analysis of Volatile Compounds

**Figure 6:**
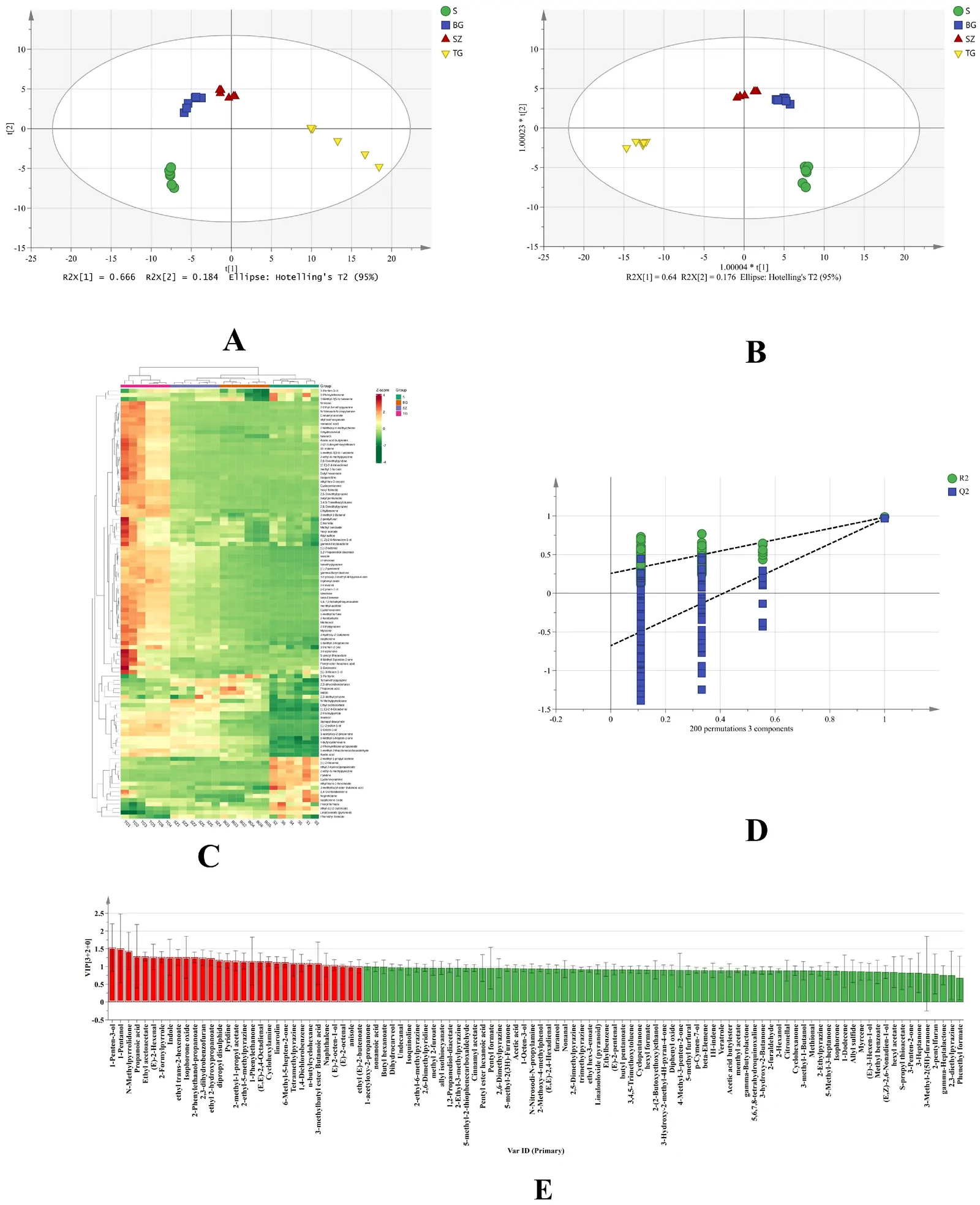
Multivariate analysis of volatile components in salt-processed *Semen Cuscutae* with different processing degrees. (**A**) PCA plot. (**B**) OPLS-DA plot. (**C**) OPLS-DA permutation test plot. (**D**) Heatmap. (**E**) VIP scores plot. (*n* = 15 per group, four groups in total. VIP, variable importance in projection; OPLS-DA, orthogonal partial least-squares discriminant analysis; PCA, principal component analysis. Variables with VIP > 1 were regarded as important contributors for sample discrimination).

Preliminary analysis of the volatile component characteristics revealed a rich diversity of volatile components detected in samples of salt-processed *Semen Cuscutae* with different processing degrees. Based on the peak volume per compound category, the clustering pattern can effectively discriminate among the sample groups. Principal component analysis (PCA) was used to process the peak volumes of 104 compounds (dependent variables). As shown in Figure 6A, the PCA model revealed significant separation among the four sample groups in the feature space: the TG group exhibited distinct heterogeneity from the SZ, S, and BG groups. While the BG and SZ groups formed independent clusters, their spatial proximity indicates that their chemical profiles are similar [25].

The results of inter-group difference modeling and validation showed that orthogonal partial least squares discriminant analysis (OPLS-DA) was further employed to quantify the differences between groups (Figure 6B). The model fitting parameters (R^2^X = 0.956, R^2^Y = 0.966, Q^2^ = 0.93) all significantly exceeded the empirical thresholds, confirming the model’s robust explanatory power and predictive efficacy. After validation through 200 permutations (Figure 6C), the Q^2^ regression line exhibited a negative intercept with the vertical axis, effectively ruling out the risk of model overfitting.

In the screening of key differential markers, variables meeting all three criteria, VIP > 1, FDR-adjusted q < 0.05, and |log2FC| ≥ 1 were defined as differential markers, and 31 differential compounds were tentatively identified (Figure 6E). Among these, 1-penten-3-ol and 1-pentanol were tentatively identified as key flavor substances, with their abundance changes showing a significant correlation with the degree of processing [26].

### 3.6. In Vitro Antioxidant Activity

**Figure 7:**
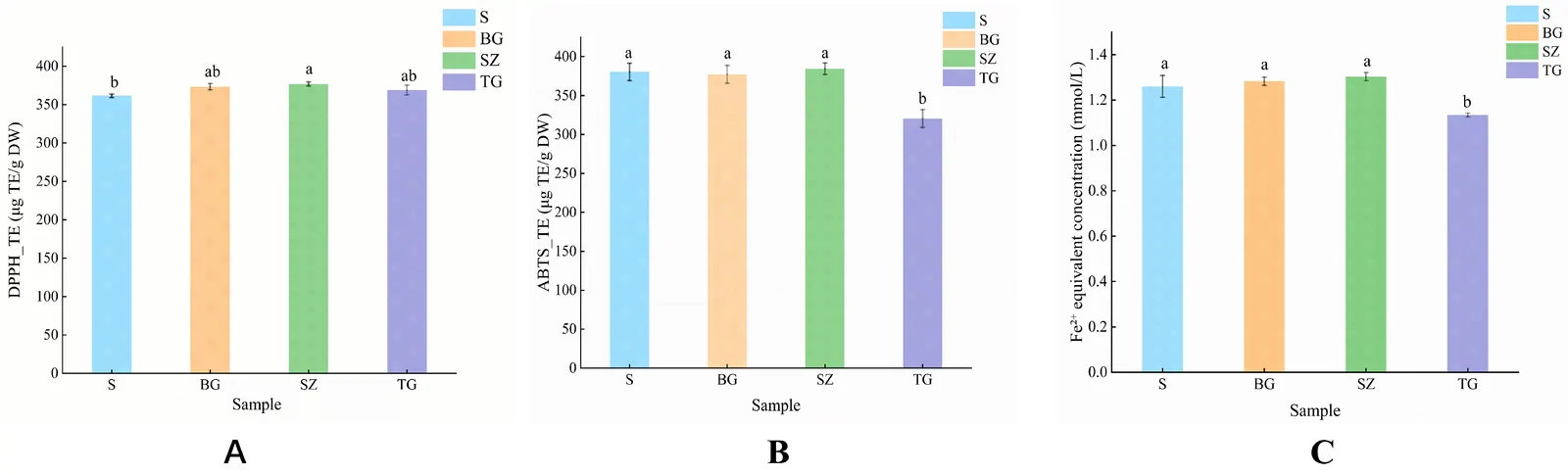
Antioxidant capacities of samples determined by DPPH (**A**), ABTS (**B**) and FRAP (**C**) assays. DPPH and ABTS results were expressed as Trolox equivalent (μmol TE/g), while FRAP result was expressed as Fe^2+^ equivalent concentration (mmol/L). Data are expressed as mean ± standard deviation (*n* = 3). One-way analysis of variance (ANOVA) followed by Tukey’s multiple comparison test. Different lowercase letters indicate significant differences at *p* < 0.05.

Three methods, including DPPH, ABTS and FRAP assays, were used to evaluate the in vitro antioxidant capacity of four groups of salt-processed *Semen Cuscutae* samples (S, BG, SZ, TG), and the results are shown in Figure 7. The results of DPPH and ABTS were expressed as Trolox equivalent (μg TE/g DW), while FRAP results were presented as Fe^2+^ equivalent concentration (mmol/L). As shown in Figure 7A, sample SZ exhibited the highest DPPH antioxidant capacity, whereas samples S and TG showed relatively lower values. Figure 7B indicated that no significant difference in ABTS antioxidant capacity was observed among S, BG and SZ groups (*p* > 0.05); the ABTS antioxidant capacity of sample TG was significantly lower than that of the above three groups (*p* < 0.05). As displayed in Figure 7C, there was no significant difference in ferric reducing capacity among S, BG and SZ samples (*p* > 0.05); the FRAP reducing capacity of sample TG was significantly lower than that of the other three groups (*p* < 0.05).

Comprehensive antioxidant assay results showed no significant differences in antioxidant activity among the S, BG, and SZ samples, whereas the TG sample exhibited significantly lower antioxidant activity than the S, BG, and SZ samples. Combined with the above HPLC quantification results, the contents of five major flavonoid components (e.g., hyperoside and astragalin) showed no significant differences among the S, BG, and SZ groups, but were significantly lower in the TG group than in the other groups. That is, the inter-group variation in antioxidant activity was generally consistent with the variation in the contents of the major flavonoid components, suggesting that flavonoids may be one of the important material bases for the in vitro antioxidant activity of *Semen Cuscutae*.

### 3.7. Multi-Source Information Integration Analysis

**Figure 8:**
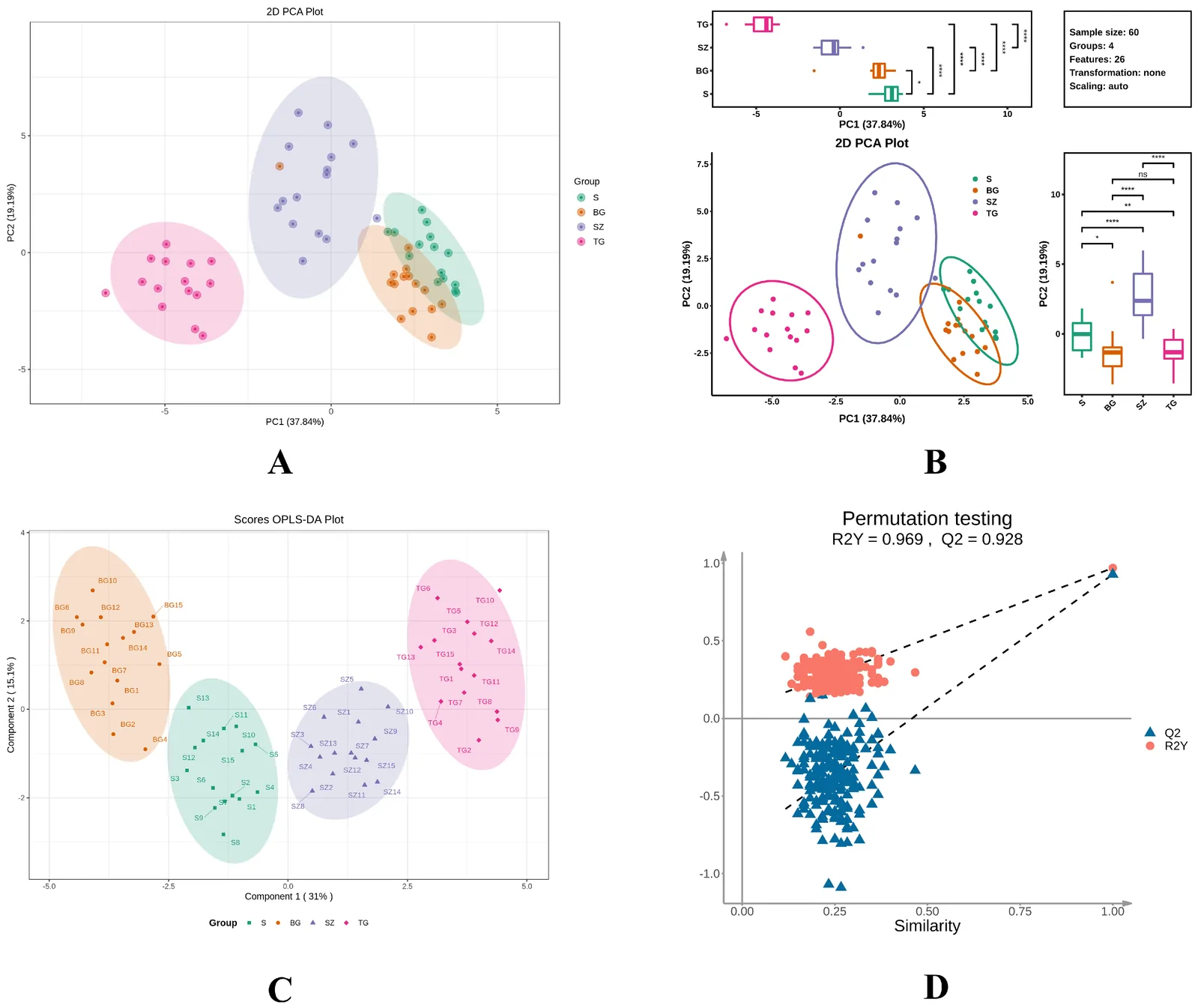
Multi-source information fusion analysis of salt-processed *Semen Cuscutae* with different processing degrees. (**A**) PCA plot. (**B**) PCA combined bar chart. (**C**) OPLS-DA plot. (**D**) OPLS-DA permutation test plot. (*n* = 15 per group, four groups in total. OPLS-DA, orthogonal partial least-squares discriminant analysis; PCA, principal component analysis; R^2^, goodness-of-fit parameter; Q^2^, predictive ability parameter * *p* < 0.05, ** *p* < 0.01, **** *p* < 0.0001, ns = not significant (*p* ≥ 0.05).

Principal component analysis (PCA) and orthogonal partial least squares discriminant analysis (OPLS-DA) were performed on four differently processed levels of salt-processed *Semen Cuscutae* by integrating multi-source information from machine vision, electronic tongue, electronic nose, and high-performance liquid chromatography (HPLC) [27]. Permutation tests were further conducted to validate the stability and reliability of the models. The results are illustrated in Figure 8 below. As shown in the PCA plot, the discriminatory effect of multidimensional information fusion between raw and under-processed samples is relatively weak, forming a trend toward clustering into one category. Overall, the sample points can be divided into three relatively independent regions, indicating that the multi-source information fusion among samples with different degrees of processing exhibits significant differences [28]. An orthogonal partial least squares discriminant analysis (OPLS-DA) model was further established. Clear separations were achieved among raw, under-processed, appropriately processed, and over-processed samples. Moreover, after validation through 200 permutation tests, R^2^Y = 0.969, Q^2^ = 0.928 were both significantly higher than the empirical thresholds, confirming the model’s strong predictive efficacy. The Q^2^ regression line exhibits a negative intercept with the vertical axis, effectively ruling out the risk of model overfitting and confirming the stability and reliability of the model [29].

### 3.8. Multi-Source Information Correlation Analysis

#### 3.8.1. Partial Least Squares Regression (PLSR) Analysis of Electronic Nose and GC-IMS

The responses of 10 electronic nose sensors (W1C, W2S, W3C, W4S, W5C, W6S, W1S, W2W, W3S, and W5S) averaged over the steady-state interval of 60–120 s were used as the X variables, and the 2-octanol-normalized relative peak volumes of 104 volatile compounds tentatively identified by GC-IMS were used as the Y variables to establish a partial least squares regression (PLSR) model. The experimental unit was the independent stir-frying batch, with 15 independent batches per group and a total of 60 independent batches. Technical replicates were first averaged within each batch and were not treated as independent samples. Samples were stratified and randomly divided by processing group into a training set of 42 batches (70%) and a prediction set of 18 batches (30%). Seven-fold cross-validation was used to determine the optimal number of latent variables, which was 2. Since technical replicates from the same batch were averaged beforehand, each batch had only one representative value; therefore, no data leakage occurred between the training and prediction sets.

**Figure 9:**
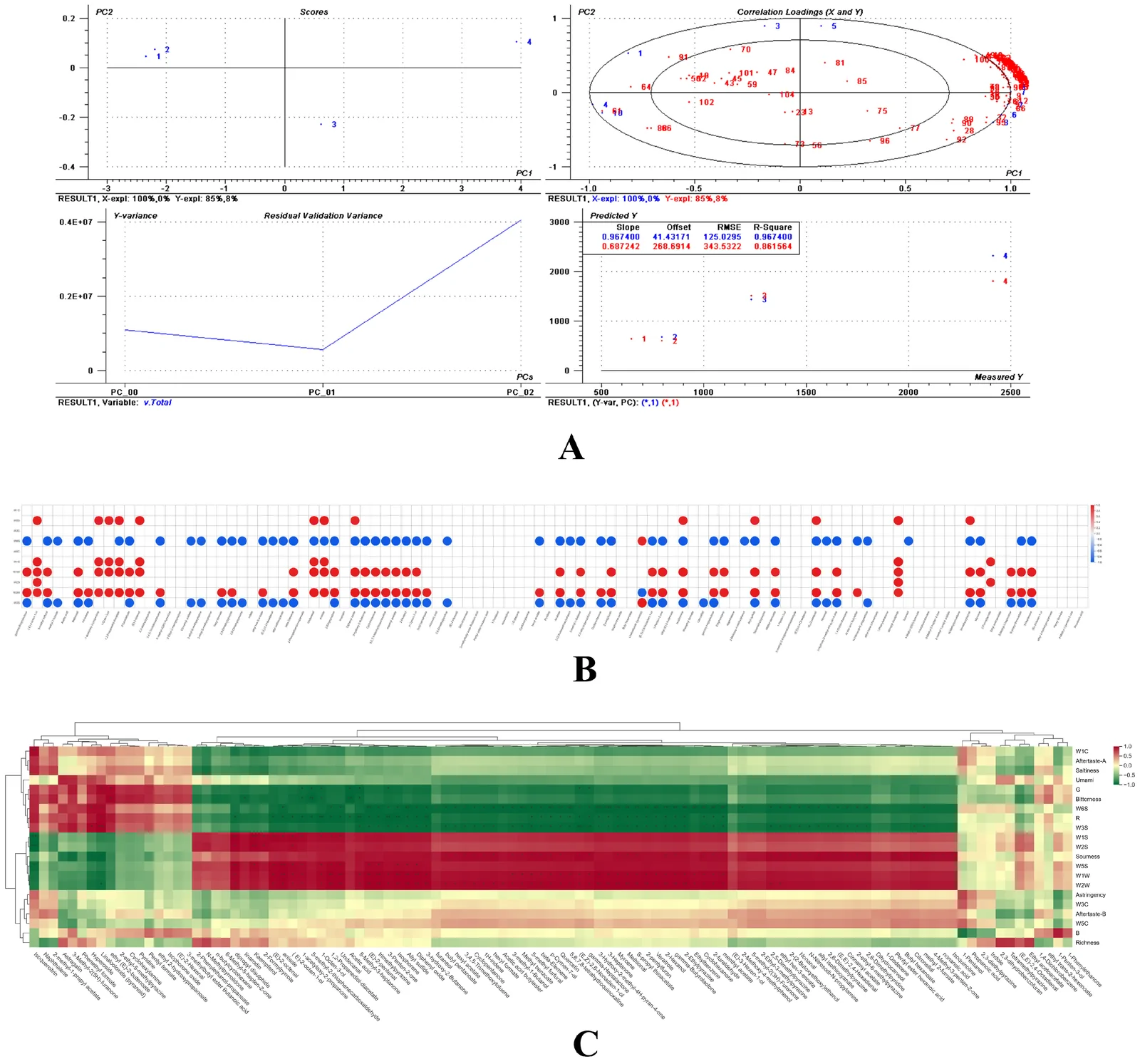
Correlation analysis. (**A**) Partial Least Squares Regression (PLSR) correlation analysis plot between electronic nose and GC-IMS data. (**B**) Multi-omics corrplot for correlation analysis between electronic nose and GC-IMS data. (**C**) Correlation analysis of biomimetic sensory technology with liquid phase intrinsic components and GC-IMS. *(n* = 15 per group, four groups in total. One-way ANOVA followed by Tukey’s HSD test, *p* < 0.05. * *p* < 0.05, ** *p* < 0.01, *** *p* < 0.001. PLSR, partial least squares regression The vertical axis represents Y-variance, with tick marks at 0.4 × 10^7^ and 0.2 × 10^7^).

The results are shown in Figure 9A. As can be seen from the loading plot, the majority of variables fall within the confidence ellipse, indicating that the model is stable, reliable, and possesses high credibility. PC1 is the primary/principal contributing axis, while PC2 has a relatively lower contribution. For this model, the slope is 0.687242, the intercept is 268.6914, the Root Mean Square Error (RMSE) is 343.5322, the coefficient of determination R^2^ (for the prediction set) is 0.861564, and R^2^ (for the fitting/training set) is 0.967400 [30]. The high R^2^ values indicate that the electronic nose signal has a good predictive capability for the GC-IMS detection results. As shown in Figure 8B, the electronic nose sensor signals (e.g., W1W, W2W) were positively correlated with the normalized peak volumes of several volatile compounds tentatively identified by GC-IMS, such as (E)-2-octenal and 3-hydroxy-2-butanone. These results indicate that the responses of electronic nose sensors are statistically correlated with a subset of volatile compounds detected by GC-IMS, providing a reference for the rapid characterization of odor fingerprints in complex systems using electronic nose. It should be noted that correlation does not imply causation, and independent validation experiments are still required to confirm whether these correlated compounds can serve as markers [31].

#### 3.8.2. Correlation Analysis of Biomimetic Sensory Technology with Liquid Phase Intrinsic Components and GC-IMS

Correlation analysis was performed among machine vision, electronic tongue, electronic nose with liquid phase intrinsic components and GC-IMS [32]. This correlation analysis was performed based on representative values from 60 independent batches, and technical replicates were excluded from statistical analysis. All *p* values were corrected using the Benjamini–Hochberg FDR method, with statistical significance defined as *p*_adj < 0.05. The detailed r values, raw *p* values, *p*_adj values and confidence intervals are provided separately in an Excel file within the Supplementary Materials due to the large volume of data. It can be seen from Figure 9C that isoquercitrin changes in the same direction as the R channel, while quercetin changes in the opposite direction to the R channel. These two compounds are tentatively identified as core flavonoid components that regulate RGB color signals. The opposite correlations of isoquercitrin and quercetin with the red channel are essentially due to the blue shift in the absorption spectrum caused by C3 glycosylation, which leads to different visible-light reflection characteristics between the two. The enhancement of the W1W/W2W signals originates from the degradation of cysteine during stir-frying, releasing H_2_S and NH_3_, which further react with Maillard reaction intermediates to generate sulfur- and nitrogen-containing volatile compounds. The greater the degree of stir-frying, the stronger this Maillard reaction pathway, and the sensor responses continuously increase, which is also consistent with the increasing trends of pyrazines and sulfur-containing compounds detected by GC-IMS. Isoquercitrin showed an extremely strong negative correlation with the W1W and W2W sensors, and a strong positive correlation with the W3S sensor. In contrast, quercetin exhibited an extremely strong positive correlation with the W1W and W2W sensors, and a strong negative correlation with the W3S and W6S sensors. These compounds serve as the key material basis mediating the responses of the odor sensors. Isoquercitrin changes in the same direction as umami intensity, and in the opposite direction to sourness intensity. Quercetin changes in the same direction as sourness, in the opposite direction to umami and bitterness, serving as the primary flavonoid component influencing the taste characteristics of Chinese herbal medicine.

Volatile components also exhibited significant correlation. Volatile ester components (e.g., ethyl (E)-2-butenoate) exhibit a strong positive correlation with the R channel. Aldehyde components (e.g., (E)-2-pentenal) and alcohol components (e.g., 1-Penten-3-ol) exhibit a strong negative correlation with the R channel. In terms of odor sensor response, ester components (e.g., 3-Methyl-2(5H)-furanone) exhibit an extremely negative correlation with the W1W and W2W sensors, and a positive correlation with the W3S and W6S sensors. Ester components (e.g., gamma-Heptalactone), ketone components (e.g., 3-Heptanone), and alcohol components (e.g., 2-Hexanol) exhibit an extremely strong positive correlation with the W1W and W2W sensors, and a strong negative correlation with the W3S and W6S sensors. In terms of taste correlation, ester components such as butyl pentanoate and ketones such as 4-Methyl-3-penten-2-one show a strong positive correlation with sourness, while showing a negative correlation with umami and bitterness. Ester components (e.g., 3-Methyl-2(5H)-furanone) show a negative correlation with sourness, while showing a positive correlation with umami and bitterness.

In summary, isoquercitrin and quercetin are flavonoid components with statistically significant correlations to indicators of biomimetic sensory technology. Esters, aldehydes, alcohols and ketones represent volatile components that are statistically associated with these biomimetic sensory indicators. These compounds can serve as candidate markers for the optimization of Chinese herbal medicine processing and sensory quality evaluation. However, their causal effects and underlying mechanisms require further verification via independent experiments. Some observed correlations may be confounded by the shared trend induced by processing intensity, and direct mechanistic relationships cannot be inferred solely from these results [33].

### 3.9. Analysis of AHP-Entropy Weight TOPSIS Method

The weight for hyperoside, astragalin, appearance character, W1W response value, W2W response value, richness response value, and the total peak intensity value of differential volatile components (VIP > 1) in flavor substances, obtained through the Analytic Hierarchy Process, were 34.4377%, 22.9974%, 14.8529%, 9.1292%, 9.1292%, 5.6729%, and 3.7807% respectively (see Table 4). The Consistency Index (CI) was 0.022, which is less than 0.1, indicating that the weight coefficients are valid. The weight coefficients calculated by the entropy-weighted TOPSIS method were 8.4293%, 8.7001%, 11.2798%, 10.8446%, 20.4710%, 27.7840%, and 12.4912% respectively. See Table 5 for the results. The detailed results of the mixed weights are shown in Table 6. The original data for the entropy-weighted TOPSIS method are provided in Table S6. The AHP-TOPSIS evaluation results are shown in Table 7. Through comprehensive assessment, it can be concluded that SZ > BG > S > TG.

### 3.10. Moisture Content of Raw and Processed Samples

As shown in Table 8, the average moisture content was 9.32% for S, 9.47% for BG, 9.30% for SZ, and 9.04% for TG. The moisture contents of all samples were generally at comparable levels. The relatively lower moisture content of TG (9.04%) was mainly attributed to the longer stir-frying duration.

## 4. Discussion

To address the current lack of objective quantitative standards for the processing endpoint of salt-processed *Semen Cuscutae*, this study constructed an evaluation method integrating multi-source information from machine vision, biomimetic sensing technologies (electronic nose and electronic tongue), and precision instrument analysis (HPLC, GC-IMS) to investigate the effects of processing degrees on the multi-dimensional qualities of “color, taste, odor, and quality” of salt-processed *Semen Cuscutae*.

The results showed that with the extension of processing time, the R and G values of salt-processed *Semen Cuscutae* decreased gradually, and the appearance color deepened progressively from gray-brown to brown. Increased processing degree elevated the responses of W1W, W5S, and W2W sensors of the electronic nose, indicating an enhanced overall odor performance, which may be associated with changes in nitrogen oxides, organic sulfides, and other compounds. However, due to the cross-sensitivity of the PEN3 sensor, the increased responses of W5S, W1W and W2W alone cannot confirm the accumulation of specific chemical components.

In terms of taste, the appropriately processed products (SZ) exhibited significantly reduced bitterness and astringency without excessive increase in sourness, presenting balanced taste attributes and a harmonious taste profile. Biomimetic sensing technologies can provide objective surrogate signals corresponding to the color, odor, and taste characteristics of samples. Nevertheless, these signals have not been verified by trained sensory panels and cannot be equivalent to human sensory evaluation or sensory preference.

Regarding the intrinsic components, the contents of hyperoside and isoquercitrin showed no significant differences among the raw, under-processed, and appropriately processed products, but decreased significantly in over-processed products. The contents of quercetin and kaempferol increased gradually with the rise in processing degree, and the over-processed group (TG) showed remarkably higher levels than the raw (S), under-processed (BG), and moderately processed (SZ) groups. This indicated that thermal processing promoted the hydrolysis of glycosidic bonds in flavonoid glycosides and the release of aglycones. However, compared with flavonoid glycosides such as hyperoside and isoquercitrin, the absolute contents of aglycones remained relatively low, which may be attributed to their further oxidation, polymerization, or matrix binding under high-temperature conditions.

The gradual color darkening of salt-processed *Semen Cuscutae* during stir-frying (reduced R and G values) is mainly caused by the Maillard reaction. Reducing sugars (glucose and fructose) in the seeds undergo carbonyl-amine reactions with amino acids (asparagine and glutamine) to form brown polymers such as melanoidins. Meanwhile, the detection of 5-methylfurfural via GC-IMS confirmed that caramelization induced by carbohydrate thermal degradation also contributes to color and flavor formation. With the increase in processing degree, flavonoid glycosides including hyperoside, isoquercitrin, and astragalin undergo glycosidic bond hydrolysis. As illustrated in Figure 4B, the continuous increase in quercetin and kaempferol contents provides direct evidence for heat-triggered glycosidic hydrolysis. Among volatile components, C6–C9 unsaturated aldehydes represented by (E)-2-octenal and (E)-2-pentenal are mainly derived from the thermal oxidation of unsaturated fatty acids (linoleic acid and linolenic acid), while pyrazines and furans are primarily Maillard reaction products. Limited by the availability of reference standards, some GC-IMS database matching results are tentative and require further verification with reference standards or GC-MS. All these changes constitute the key biochemical basis for the color, odor, and chemical composition evolution of salt-processed *Semen Cuscutae* during stir-frying.

Notably, the stir-frying conditions adopted in this study belonged to short-term high-temperature inactivation treatment, which exceeded the threshold for maintaining seed viability and enzyme activity. Under such conditions, seed cell structures are destroyed, and endogenous protein enzymes including amylase, lipase, SOD, POD, and CAT undergo irreversible thermal denaturation and inactivation. Therefore, these physiological indices were not investigated in this study. Odor variations are mainly attributed to the thermal oxidation and cleavage of linoleic acid and linolenic acid, generating C6–C9 aldehydes and ketones such as (E)-2-octenal and (E)-2-pentenal, which is consistent with the elevated responses of W1W, W5S, and W2W sensors under intensified processing. Flavonoid changes reflect the thermal stability and chemical transformation of these small-molecule antioxidants (e.g., glycoside hydrolysis for aglycone release), rather than variations in enzymatic antioxidant systems. Accordingly, this study focuses on the variation patterns and biochemical mechanisms of small-molecule chemical components under thermal treatment, instead of the physiological responses of viable seeds.

Existing literature indicates that after salt-processing and wine-processing, the total flavonoid content of *Semen Cuscutae* is higher than that of the raw products, and both processing methods can promote the dissolution of multiple components such as hyperoside and quercetin [34]. Based on this, this study further demonstrates that hyperoside and isoquercitrin contents drop markedly due to excessive processing, indicating that the control of processing time is important for the levels of intrinsic components. The results of this study showed that the contents of flavonoids such as hyperoside and astragalin in appropriately processed salt-processed *Semen Cuscutae* remained at relatively high levels. Moreover, the comprehensive evaluation using AHP-TOPSIS yielded the optimal result, indicating that appropriate processing is beneficial for preserving the intrinsic active components. The AHP–entropy weight–TOPSIS model established in this study is exploratory and has not been validated with independent samples; its generalizability requires further verification. This result is consistent with the understanding of “processing to moderation” in traditional experience. However, this study lacks clinical or sensory verification and thus cannot objectively validate its rationality. Existing literature indicates that flavonoids such as hyperoside and astragalin serve as the potential material basis for the effects of “salt-processing guiding the medicine to the kidney” and fetus-stabilizing of salt-processed *Semen Cuscutae*. This study only verifies that the contents of these constituents change with the processing degree, and relatively high levels of the above components are maintained in moderately processed samples, demonstrating that moderate processing is conducive to retaining these potential active markers. Focusing on the chemical composition changes during processing, the present work did not perform biological activity or in vivo pharmacodynamic tests, and the study was not designed to validate this material basis hypothesis. Hyperoside is the quality-control marker for *Cuscutae Semen* specified in the 2025 edition of the Chinese Pharmacopoeia (no less than 0.10%). Hyperoside and astragalin are both characteristic flavonoids of Cuscuta australis and can be used to distinguish the adulterant Cuscuta japonica. They undergo regular hydrolysis during processing, with a corresponding increase in aglycones, thus sensitively reflecting the degree of processing.

Modern studies have shown that *Semen Cuscutae* exhibits the effects of nourishing the liver and kidney, arresting seminal emission and reducing urination, stabilizing the fetus, improving eyesight, and relieving diarrhea [35]. Flavonoids are the main pharmacodynamic material basis of *Semen Cuscutae* [36]. Previous studies by our research group have found that the enhanced efficacy of salt-processed *Semen Cuscutae* is attributed to the synergistic interaction of flavonoids, phenolic acids, and other components, which collectively exert the effects of tonifying the kidney and stabilizing the fetus [37].

Studies [38,39] have confirmed that the aforementioned flavonoids are closely associated with the estrogen-like effects and anti-abortive activity of *Semen Cuscutae.* Therefore, maintaining stable contents of these components is of great significance for ensuring the quality consistency of salt-processed *Semen Cuscutae* when used as a functional food ingredient.

This study systematically elucidated the influence patterns of different processing degrees on its multi-dimensional qualities. However, the current study did not cover samples from all producing areas or samples with different storage years. The established evaluation model and marker thresholds require further external validation before being applied to other producing areas and industrial production batches. On this basis, future research can expand the sample size, develop portable online rapid detection equipment, and extend this evaluation model to real-time quality monitoring during the production of functional food ingredients.

## 5. Conclusions

In this study, HPLC and GC-IMS techniques were employed to systematically investigate the effects of four distinct processing degrees on the intrinsic and volatile components of salt-processed *Semen Cuscutae*. Multivariate statistical analysis showed that different processing degrees enabled the discrimination of five flavonoid components in salt-processed *Semen Cuscutae*, and 31 differential volatile compounds were screened. This indicates that processing degree exerted a significant influence on its intrinsic components and volatile profile. Combined with bionic sensory technology, we further performed digital characterization of the morphological appearance of salt-processed *Semen Cuscutae* and tentatively identified significant differences in its color, odor, and taste across different processing degrees. Isoquercitrin and quercetin were tentatively identified as the core flavonoid components that exhibited significant correlations with RGB values, electronic nose response signals, and taste indicators. Esters, aldehydes, alcohols, and ketones were key volatile components showing significant correlations with indicators obtained from bionic sensory technology. These volatile compounds can serve as key targets for process optimization and sensory quality evaluation of traditional Chinese medicine. Electronic nose sensors (e.g., W1W, W2W) showed strong correlations with key volatile components tentatively identified by GC-IMS (e.g., (E)-2-Octenal), suggesting an association between sensor responses and odor changes during processing. Finally, the AHP-TOPSIS model was adopted to conduct multidimensional quality evaluation of salt-processed *Semen Cuscutae* with four different processing degrees based on bionic sensory characteristics, HPLC characteristic components and volatile components determined by GC-IMS. Combined with three in vitro antioxidant assays (DPPH, ABTS and FRAP), the results showed that the appropriately processed group (SZ) exhibited better overall performance than the raw group (S), under-processed group (BG) and over-processed group (TG). By integrating bionic sensory data, chemical profiles and in vitro bioactivity, this study realized a multimodal comprehensive evaluation covering the “color, odor, taste, intrinsic quality and bioactivity” of salt-processed *Semen Cuscutae*. It provides theoretical support for the optimization of processing parameters and comprehensive quality assessment of salt-processed *Semen Cuscutae*.

## Figures and Tables

**Table 1:** Regression equations of calibration curves.

Compound	Regression Equation	R^2^	Linear Range (μg/mL)
hyperoside	Y = 0.3407X + 1.2439	0.9997	5.2841–422.4077
astragalin	Y = 0.3084X + 0.1405	0.9996	2.1255–169.4490
isoquercitrin	Y = 0.4401X + 0.5277	0.9997	1.5614–125.0478
quercetin	Y = 0.5601X − 0.0114	0.9998	0.1525–12.2217
kaempferol	Y = 0.5844X − 0.0037	0.9993	0.1914–15.4925

**Table 2:** Results of method validation (LOD, S/N = 3; LOQ, S/N = 10).

Compound	LOD (μg/mL)	LOQ (μg/mL)	Precision RSD (%)
hyperoside	1.25	4.01	1.67
astragalin	0.64	2.01	1.16
isoquercitrin	0.31	1.04	1.66
quercetin	0.03	0.09	1.74
kaempferol	0.03	0.11	1.60

**Table 3:** Results of method validation.

Sample	Compound	Stability RSD (%)	Repeatability RSD (%)	Average Recovery (%)	RSD of Recovery Test (%)
S	hyperoside	1.30	1.84	99.78	1.43
	astragalin	1.33	2.58	102.07	1.75
	isoquercitrin	1.69	2.62	95.27	2.06
	quercetin	2.76	2.89	93.21	3.28
	kaempferol	2.66	2.73	102.62	1.69
BG	hyperoside	1.44	2.12	97.64	1.67
	isoquercitrin	1.65	2.74	98.61	2.11
	astragalin	1.50	2.88	99.67	1.98
	quercetin	2.54	2.96	101.24	2.74
	kaempferol	2.43	3.01	96.43	2.23
SZ	hyperoside	2.71	1.99	98.67	3.11
	isoquercitrin	1.56	2.54	95.43	2.56
	astragalin	1.67	1.77	94.32	2.45
	quercetin	1.98	2.02	101.33	1.89
	kaempferol	2.02	1.98	93.51	1.74
TG	hyperoside	2.45	3.34	97.65	3.01
	isoquercitrin	2.34	3.45	94.78	2.98
	astragalin	2.65	2.11	98.89	1.77
	quercetin	1.77	2.47	98.54	1.64
	kaempferol	1.64	2.65	99.43	2.67

**Table 4:** AHP analytic hierarchy process results. (The contents of hyperoside, isoquercitrin, astragalin, quercetin, and kaempferol are all expressed in %).

	Priority Vector	ωAHP	Maximum Eigenvalue		CI Value
Hyperoside	2.411	34.4377%			
Astragalin	1.610	22.9974%	7.131	0.022	
Appearance character	1.040	14.8529%			
W1W	0.639	9.1292%			
W2W	0.639	9.1292%			
Richness	0.397	5.6729%			
Flavor substance	0.265	3.7807%			

**Table 5:** Entropy weight TOPSIS weight calculation results. (The contents of hyperoside, isoquercitrin, astragalin, quercetin, and kaempferol are all expressed in %).

	Information Entropy Value (e)	Information Utility Value (d)	ωEW-TOPSIS
Hyperoside	0.8059	0.1941	8.4293%
Astragalin	0.7996	0.2004	8.7001%
Appearance character	0.7402	0.2598	11.2798%
W1W	0.7503	0.2497	10.8446%
W2W	0.5286	0.4714	20.4710%
Richness	0.3602	0.6398	27.7840%
Flavor substance	0.7123	0.2877	12.4912%

**Table 6:** AHP-entropy weight TOPSIS mixture weighted weight. (The contents of hyperoside, isoquercitrin, astragalin, quercetin, and kaempferol are all expressed in %).

	AHP	EW-TOPSIS	Mixed Weights ω
Hyperoside	34.4377%	8.4293%	25.2928%
Astragalin	22.9974%	8.7001%	17.4326%
Appearance character	14.8529%	11.2798%	14.5973%
W1W	9.1292%	10.8446%	8.6261%
W2W	9.1292%	20.4710%	16.2763%
Richness	5.6729%	27.7840%	13.6798%
Flavor substance	3.7807%	12.4912%	4.09522%

**Table 7:** AHP-entropy weight TOPSIS evaluation results.

Item	Positive I.	Negative Ideal Solution Distance D-	Relative Closeness C	Ranking Result
S	0.757	0.582	0.435	3
BG	0.64	0.591	0.48	2
SZ	0.272	0.869	0.762	1
TG	0.774	0.571	0.425	4

**Table 8:** Determination results of moisture content in raw and processed samples.

Number	Water Content (%) S	Water Content (%) BG	Water Content (%) SZ	Water Content (%) TG
31-1	9.55	9.83	9.05	9.05
31-2	9.52	10.01	10.10	9.92
32-1	9.94	10.39	9.93	9.87
32-2	9.87	9.59	9.20	9.30
33-1	9.80	10.27	9.35	9.56
33-2	9.86	10.36	10.14	9.18
34-1	9.96	9.89	9.65	9.50
34-2	9.67	10.22	10.27	9.97
35-1	9.79	10.15	9.40	8.89
35-2	9.73	9.59	9.97	8.78
36-1	9.02	8.98	9.39	9.35
36-2	9.14	9.88	8.48	9.54
37-1	9.89	9.27	9.68	9.91
37-2	9.83	8.78	9.73	9.92
38-1	9.61	8.56	8.49	8.93
38-2	9.91	9.25	9.02	8.53
39-1	9.72	9.38	8.81	8.16
39-2	9.78	9.17	9.95	8.45
40-1	9.27	9.76	8.95	9.72
40-2	9.17	9.81	9.04	8.21
41-1	8.95	9.92	9.82	8.78
41-2	8.92	8.68	8.74	8.98
42-1	8.67	8.75	9.62	8.16
42-2	8.66	9.58	8.42	8.19
43-1	8.47	8.70	9.41	8.97
43-2	8.38	8.95	9.49	8.07
44-1	8.09	8.76	8.85	9.03
44-2	8.09	8.68	8.66	8.96
45-1	9.15	9.89	8.88	8.95
45-2	9.11	8.89	8.58	8.33
Average	9.32	9.47	9.30	9.04

## Data Availability

The datasets from the current study are available from the corresponding author on reasonable request.

## Supplementary Materials

The following supporting information can be downloaded at: https://www.mdpi.com/article/10.3390/foods15193570/s1, Table S1: Performance description of PEN3 electronic nose sensors (The performance descriptions above represent the nominal target analytes provided by the instrument manufacturer. Owing to the inherent cross sensitivity of metal‑oxide sensors in the PEN3 electronic nose, changes in a single sensor signal cannot provide definitive, compound‑specific identification of volatile substances in salt processed *Semen Cuscutae*). Table S2: E-tongue System Sensor Type and Performance Description. Table S3: Evaluation Criteria for salt-processed *Semen Cuscutae* Appearance Character. Table S4: Total Scores of Appearance Characteristics for Different Processed degrees salt-processed *Semen Cuscutae.* Table S5: Judgment Priority Matrix for Pairwise Comparison of Indicators. Table S6: Raw Data From AHP-entropy weight TOPSIS. (The contents of hyperoside and astragalin are expressed in %). Table S7: Tentatively identified results of some VOCs in salt-processed *Cuscuta australis* R. Br under different processing methods. Table S8: Detailed information of the 15 batches of raw *Semen Cuscutae*. Table S9: System suitability results of the five analytes. Table S10: The contents of five flavonoid components in each salt-processed sample (Values are expressed as g/g). Table S11: Volatile organic compounds (VOCs) with corresponding retention times, Drift time and peak volumes.

## Abbreviations

AHP-TOPSIS, Analytic Hierarchy Process-Technique for Order Preference by Similarity to Ideal Solution; B, Blue channel; BG, Under processed products; E-nose, Electronic nose; E-tongue, Electronic tongue; GC-IMS, Gas Chromatography–Ion Mobility Spectrometry; G, Green channel; HPLC, High-Performance Liquid Chromatography; R, Red channel; S, Raw products; SZ, Appropriately processed products; TG, Over processed products.
