# Supplementary material for: Multi-Source Data Fusion Integrating HPLC, GC-IMS, and Multidimensional Biomimetic Technologies for Evaluating the Effects of Different Processing Degrees on the Quality of Salt-Processed Semen Cuscutae
Source: Foods. 2026 Oct 8;15(19):3570. doi: 10.3390/foods15193570 (PMC13665127; doi:10.3390/foods15193570)
Supplement: Supplementary file 1 [file foods-15-03570-s001.zip › foods-4555513-Supplementary Tables.pdf]

Table S1 Performance description of PEN3 electronic nose sensors (The performance descriptions above represent the nominal target analytes provided by the instrument manufacturer. Owing to the inherent cross-sensitivity of metal-oxide sensors in the PEN3 electronic nose, changes in a single sensor signal cannot provide definitive, compound-specific identification of volatile substances in salt-processed *Semen Cuscutae*.

Array number	Sensor name	Performance description
1	W1C	Aromatic compounds, benzene-type substances
2	W5S	High sensitivity, nominally responsive to nitrogen-containing oxides
3	W3C	Ammonia, nominally sensitive to aromatic compounds
4	W6S	Mainly selective to hydrogen
5	W5C	Alkane-type aromatic compounds
6	W1S	Sensitive to short-chain alkanes such as methane
7	W1W	Nominally responsive to inorganic sulfides
8	W2S	Sensitive to alcohols, ethers, aldehydes and ketones
9	W2W	Aromatic components, nominally responsive to organic sulfides
10	W3S	Sensitive to alkanes, long-chain alkane species

Table S2 E-tongue System Sensor Type and Performance Description

Array number	Sensor name	Performance description
1	AAE	Umami
2	CT0	Saltiness
3	CA0	Sourness
4	C00	Bitterness
5	AE1	Astringency
6	AAE2	Richness
7	AAE3	Aftertaste-A
8	AAE4	Aftertaste-B

Table S3 Evaluation Criteria for salt-processed *Semen Cuscutae* Appearance Character

Color	Score	Aroma	Score	State	Score
Greyish-brown	1	No aroma	1	No bursting	1
Tan	5	Slight aroma	5	Occasional bursting	5
Brown	3	Distinct aroma	3	Distinct bursting	3

Table S4 Total Scores of Appearance Characteristics for Different Processed degrees salt-processed *Semen Cuscutae*

Grouping	Color	Aroma	State	Total Scores
S	1	1	1	3
BG	1	5	1	7
SZ	5	5	5	15
TG	3	3	3	9

Table S5 Judgment Priority Matrix for Pairwise Comparison of Indicators

	Hyperoside	Astragalin	Appearance character	W1W	W2W	Richness	Flavor substance
Hyperoside	1	2	3	4	4	5	6
Astragalin	0.5	1	2	3	3	4	5
Appearance character	0.333	0.5	1	2	2	3	4
W1W	0.25	0.333	0.5	1	1	2	3
W2W	0.25	0.333	0.5	1	1	2	3
Richness	0.2	0.25	0.333	0.5	0.5	1	2
Flavor substance	0.167	0.2	0.25	0.333	0.333	0.5	1

Table S6 Raw Data From AHP-entropy weight TOPSIS. (The contents of hyperoside and astragalin are expressed in %.)

	Hyperoside	Astragalin	Appearance character	W1W	W2W	Richness	Flavor substance
S	0.942	0.178	3	3.18457	1.62401	0.27222	36251.14675
BG	0.924	0.181	7	4.18457	1.65882	1.46422	33868.76470
SZ	0.924	0.189	15	5.18457	2.22944	8.45333	41064.35173
TG	0.708	0.153	9	6.18457	3.13109	0.30911	44663.46074

Table S7 Tentatively identified results of some VOCs in salt-processed *Cuscuta australis* R. Br under different processing methods

Category (Relative content %)	No.	Name	Element composition	Molecular weight	Retention index	Retention time /s	Migration time/ms
Esters (25.51%)	1	gamma-Butyrolactone	C4H6O2	86.1	1614.6	1198.657	1.08525
	2	methyl 2-furoate	C6H6O3	126.1	1545.1	1046.924	1.47639
	3	5-methyl-2(3H)-Furanone	C5H6O2	98.1	1430.8	838.116	1.12183
	4	butyl pentanoate	C9H18O2	158.2	1296.6	645.425	1.40446
	5	gamma-Heptalactone	C6H10O2	114.1	1146.9	399.791	1.52859
	6	1-acetyloxy-2-propanone	C5H8O3	116.1	1483.1	927.91	1.10029
	7	1,2-Propanediol-diacetate	C7H12O4	160.2	1473.8	911.335	1.22134
	8	Cinnamyl acetate	C11H12O2	176.2	1397.1	784.867	1.08243
	9	hexyl formate	C7H14O2	130.2	1365.6	738.178	1.32682
	10	ethyl (E)-hex-3-enoate	C8H14O2	142.2	1330.4	689.275	1.254
	11	2-phenylethyl propanoate	C11H14O2	178.2	1324.8	681.834	1.34547
	12	3-methylbutyl butanoate	C9H18O2	158.2	1274.1	602.926	1.38782
	13	pentyl hexanoate	C11H22	186.3	1269.	593.53	1.53805

			O2		2	1	
	14	hexyl acetate	C8H16O2	144.2	1225.1	515.266	1.41251
	15	Butyl hexanoate	C10H20O2	172.3	1188.6	458.074	1.46019
	16	ethyl (E)-but-2-enoate	C6H10O2	114.1	1148.7	402.117	1.18372
	17	Phenethyl formate	C9H10O2	150.2	1145.4	397.913	1.28564
	18	Methyl benzoate	C8H8O2	136.1	1106.6	350.484	1.20802
	19	butyl acetate	C6H12O2	116.2	1069.1	313.317	1.23729
	20	ethyl (E)-hex-2-enoate	C8H14O2	142.2	1069.1	313.238	1.32145
	21	isobutyl acetate	C6H12O2	116.2	1018.5	270.427	1.23355
	22	Ethyl acetoacetate	C6H10O3	130.1	935	223.818	1.14913
	23	S-propyl thioacetate	C5H10OS	118.2	869.7	195.714	1.19775
	24	ethyl 2-hydroxypropanoate	C5H10O3	118.1	792.4	166.938	1.13878
	25	Pentyl formate	C6H12O2	116.2	802.5	170.438	1.28089
	26	menthyl acetate	C12H22O2	198.3	1291.8	638.151	1.23353
	27	3-Methyl-2(5H)-furanone	C5H6O2	98.1	1042.6	290.017	1.11146
Alcohols (12.24%)	28	(E)-2-octen-1-ol	C8H16O	128.2	1610.4	1189.052	1.15454

	29	1-Octen-3-ol	C8H16O	128.2	1477.9	918.541	1.15988
	30	2-Hexanol	C6H14O	102.2	1291.4	637.382	1.27958
	31	p-cymen-7-ol	C10H14O	150.2	1291.4	637.5	1.33095
	32	Dihydrocarveol	C10H18O	154.3	1273.2	601.218	1.31945
	33	1-Pentanol	C5H12O	88.1	1260.4	577.045	1.51083
	34	2-(2-Butoxyethoxy)ethanol	C8H18O3	162.2	1213.5	496.391	1.31447
	35	3-methylbutan-1-ol	C5H12O	88.1	1213.3	496.09	1.48758
	36	(E,Z)-2,6-Nonadien-1-ol	C9H16O	140.2	1168.5	429.018	1.17707
	37	1-Penten-3-ol	C5H10O	86.1	1106.4	350.292	1.3501
	38	(E)-3-Hexen-1-ol	C6H12O	100.2	834.7	182.104	1.23896
Ketones (12.24%)	39	3-hydroxybutan-2-one	C4H8O2	88.1	1292.8	640.339	1.05905
	40	Cyclohexanone	C6H10O	98.1	1292.1	638.861	1.15647
	41	Cyclopentanone	C5H8O	84.1	1192.8	464.498	1.10542
	42	Isophorone oxide	C9H14O2	154.2	1192.2	463.63	1.33342
	43	pent-3-en-2-one	C5H8O	84.1	1154.9	410.355	1.0685
	44	Isophorone	C9H14O	138.2	1146.	399.42	1.25795

					6	6	
	45	1-Phenylethanone	C8H8O	120.2	1060.4	305.504	1.18015
	46	6-Methyl-5-hepten-2-one	C8H14O	126.2	1021.3	272.672	1.18078
	47	5-Methyl-3-heptanone	C8H16O	128.2	943.4	227.724	1.27784
	48	3-Heptanone	C7H14O	114.2	874.6	197.659	1.22475
	49	4-Methyl-3-penten-2-one	C6H10O	98.1	763.6	157.346	1.12244
Aldehydes (11.22%)	50	5-methylfurfural	C6H6O2	110.1	1578.2	1116.79	1.12918
	51	Methional	C4H8OS	104.2	1505.3	968.98	1.08704
	52	furfural	C5H4O2	96.1	1467.9	900.986	1.08302
	53	(E)-2-octenal	C8H14O	126.2	1470.3	905.067	1.33309
	54	(E,E)-2,4-Hexadienal	C6H8O	96.1	1347.4	712.475	1.45037
	55	Undecanal	C11H22O	170.3	1329.9	688.6	1.59604
	56	(E)-2-Hexenal	C6H10O	98.1	1274.7	604.207	1.18362
	57	5-methyl-2-thiophenecarboxaldehyde	C6H6OS	126.2	1097.7	340.522	1.17322
	58	(E,E)-2,4-Octadienal	C8H12O	124.2	1093.1	335.906	1.26507
	59	(E)-2-pentenal	C5H8O	84.1	1092.	335.66	1.36123

					8	5	
	60	Nonanal	C9H18O	142.2	1095.3	338.119	1.47357
Aromatic hydrocarbons (11.22%)	61	1H-indene	C9H8	116.2	1505.6	969.499	1.11836
	62	3,4,5-Trimethoxytoluene	C10H14O3	182.2	1432.6	841.013	1.41825
	63	anisole	C7H8O	108.1	1311.4	664.364	1.05376
	64	linarodin	C9H10O2	150.2	1309.5	661.836	1.22827
	65	diphenyl ether	C12H10O	170.2	1307.7	659.574	1.29484
	66	Veratrole	C8H10O2	138.2	1222.4	510.698	1.57719
	67	Ethylbenzene	C8H10	106.2	1136.9	386.987	1.07545
	68	Naphthalene	C10H8	128.2	1135.6	385.389	1.11559
	69	2-Methoxy-4-methylphenol	C8H10O2	138.2	1133.3	382.421	1.1827
	70	1,4-Dichlorobenzene	C6H4Cl2	147	1076.6	320.21	1.12863
Terpenoids (4.08%)	71	linalool oxide (pyranoid)	C10H18O2	170.3	1182.6	449.218	1.26266
	72	β-elemene	C15H24	204.4	1329.7	688.375	1.43754
	73	Citronellal	C10H18	154.3	1148.	401.53	1.38148

			O		2	9	
	74	Myrcene	C10H16	136.2	994.1	252.746	1.28843
Carboxylic acids (3.06%)	75	Acetic acid	C2H4O2	60.1	1492.1	944.36	1.05139
	76	nonanoic acid	C9H18O2	158.2	1291	636.554	1.55059
	77	Propanoic acid	C3H6O2	74.1	733.4	147.883	1.10041
Miscellaneous (20.41%)	78	allyl isothiocyanate	C4H5NS	99.2	1334.8	695.237	1.36144
	79	Indole	C8H7N	117.2	1338.5	700.293	1.14864
	80	2,3-diethylpyrazine	C8H12N2	136.2	1438.7	851.135	1.22686
	81	2-Ethyl-3-methylpyrazine	C7H10N2	122.2	1400.4	789.999	1.16553
	82	2-ethyl-6-methylpyrazine	C7H10N2	122.2	1388.6	772.094	1.17293
	83	2-ethyl-5-methylpyrazine	C7H10N2	122.2	1384.4	765.777	1.19996
	84	2,5-Dimethylpyrazine	C6H8N2	108.1	1338.9	700.818	1.11576
	85	2,6-Dimethylpyrazine	C6H8N2	108.1	1333.7	693.772	1.13636
	86	2-Ethylpyrazine	C6H8N2	108.1	1309.4	661.703	1.15261
	87	5,6,7,8-tetrahydroquinoxaline	C8H10N2	134.2	1292.1	638.801	1.20253

88	Tetramethylpyrazine	C8H12N2	136.2	1124.8	371.976	1.21099
89	trimethylpyrazine	C7H10N2	122.2	984.9	247.986	1.1695
90	2,6-Dimethylpyridine	C7H9N	107.2	1277.9	610.312	1.08506
91	Pyridine	C5H5N	79.1	1213.6	496.542	1.24553
92	Isoquinoline	C9H7N	129.2	1251.3	560.356	1.19785
93	2,3-dihydrobenzofuran	C8H8O	120.2	1208.4	488.286	1.09952
94	2-pentylfuran	C9H14O	138.2	1188.9	458.595	1.25283
95	3-Hydroxy-2-methyl-4H-pyran-4-one	C6H6O3	126.1	1091.1	333.975	1.5257
96	furaneol	C6H8O3	128.1	1054	299.815	1.20106
97	N-Methylpyrrolidone	C5H9NO	99.1	1020.8	272.251	1.10727
98	2-Formylpyrrole	C5H5NO	95.1	989.7	250.427	1.37804
99	1-Dodecene	C12H24	168.3	1251.1	560.144	1.42767
100	Cyclohexylamine	C6H13N	99.2	1227.1	518.466	1.18161
101	Allyl sulfide	C6H10S	114.2	1134.2	383.505	1.3268
102	N-Nitrosodi-N-propylamine	C6H14N2O	130.2	1069.4	313.571	1.27185
103	dipropyl disulfide	C6H14S	150.3	1059.	304.85	1.4701

		2		7	4	
104	n-butylcyclohexane	C10H20	140.3	1042.2	289.74	1.25385

Table S8 Detailed information of the 15 batches of raw *Semen Cuscutae.*

No.	Herb Name	Origin	Weight/kg	Source	Batch No.
1	*Semen Cuscutae*	Inner Mongolia	2	Bozhou Traditional Chinese Medicine Professional Trading Market	20230608031
2	*Semen Cuscutae*	Inner Mongolia	2	Bozhou Traditional Chinese Medicine Professional Trading Market	20230608032
3	*Semen Cuscutae*	Inner Mongolia	2	Bozhou Traditional Chinese Medicine Professional Trading Market	20230608033
4	*Semen Cuscutae*	Inner Mongolia	2	Bozhou Traditional Chinese Medicine Professional	20230608034

				Trading Market	
5	*Semen Cuscutae*	Inner Mongolia	2	Bozhou Traditional Chinese Medicine Professional Trading Market	20230608035
6	*Semen Cuscutae*	Inner Mongolia	2	Bozhou Traditional Chinese Medicine Professional Trading Market	20230608036
7	*Semen Cuscutae*	Liaoning	2	Bozhou Traditional Chinese Medicine Professional Trading Market	20230608037
8	*Semen Cuscutae*	Liaoning	2	Bozhou Traditional Chinese Medicine Professional Trading Market	20230608038
9	*Semen Cuscutae*	Liaoning	2	Bozhou Traditional Chinese Medicine Professional Trading Market	20230608039
10	*Semen Cuscutae*	Liaoning	2	Bozhou Traditional Chinese Medicine	20230608040

				Professional Trading Market	
11	*Semen Cuscutae*	Ningxia	2	Bozhou Traditional Chinese Medicine Professional Trading Market	20230608041
12	*Semen Cuscutae*	Ningxia	2	Bozhou Traditional Chinese Medicine Professional Trading Market	20230608042
13	*Semen Cuscutae*	Ningxia	2	Bozhou Traditional Chinese Medicine Professional Trading Market	20230608043
14	*Semen Cuscutae*	Ningxia	2	Bozhou Traditional Chinese Medicine Professional Trading Market	20230608044
15	*Semen Cuscutae*	Ningxia	2	Bozhou Traditional Chinese Medicine Professional Trading Market	20230608045

Table S9 System suitability results of the five analytes

Analyte		Retention time/min	Resolution	Number of theoretical plates
Hyperoside	mixed standard	20.927	2.63	62090
Isoquercitrin		21.8	14.42	69362
Astragalin		27.27	1.97	95704
Quercetin		48.023	31.59	66972
Kaempferol		66.22	4.07	411643
Hyperoside	S	21.06	2.57	61691
Isoquercitrin		21.923	15.14	66581
Astragalin		27.41	1.94	83608
Quercetin		48.357	29.84	72125
Kaempferol		66.38	3.96	415235
Hyperoside	BG	21.103	2.58	63858
Isoquercitrin		21.977	15.06	68037
Astragalin		27.297	2.14	77013
Quercetin		48.297	18.98	77376
Kaempferol		66.343	2.86	487041
Hyperoside	SZ	21.053	2.58	63377
Isoquercitrin		21.887	14.89	66844
Astragalin		27.377	2.55	86606
Quercetin		48.303	19.21	72023
Kaempferol		66.353	4.06	452382
Hyperoside		21.03	2.56	60790
Isoquercitrin		21.893	15.29	68364
Astragalin		27.363	2.64	88284

Quercetin	TG	48.253	18.56	73210
Kaempferol		66.363	5.01	475019

Table S10 The contents of five flavonoid components in each salt-processed sample （Values are expressed as g/g)

Sample	Hyperoside	Isoquercitrin	Astragalin	Quercetin	Kaempferol
S1	0.008026552	0.001435934	0.001969536	0.000211815	0.000554003
S2	0.011697138	0.001333904	0.000561145	0.000157568	0.000162364
S3	0.006942144	0.001529538	0.001979245	0.000111159	0.000844707
S4	0.008341794	0.001656717	0.002596767	0.000176765	0.000868733
S5	0.007632461	0.001347835	0.002154728	0.000239092	0.001872605
S6	0.007251603	0.001841942	0.00373024	0.000186445	0.000949996
S7	0.012641899	0.00146115	0.000523435	0.000230847	0.000205019
S8	0.013042412	0.001828576	0.000716658	0.000235011	0.000165496
S9	0.013494384	0.001824645	0.000750579	0.000124326	0.000138725
S10	0.009490486	0.001739144	0.002120293	0.000195206	0.000496695
S11	0.007400962	0.001612372	0.002573383	0.000376765	0.001608505
S12	0.00842241	0.00137597	0.001964477	0.000254999	0.001428915

	7	6			
S13	0.007452045	0.001608949	0.002199168	0.000133058	0.000534687
S14	0.011520421	0.001596219	0.000537268	0.000183566	0.0098556376781888
S15	0.007965646	0.001636383	0.00239114	0.000245627	0.001116003
BG1	0.00615534	0.001560339	0.002053602	0.000317374	0.000493091
BG2	0.011100386	0.001320632	0.000443074	0.000216268	0.00011666
BG3	0.007993246	0.001798127	0.002136125	0.00021398	0.00082686
BG4	0.007240419	0.00140438	0.001776431	0.000189149	0.000557357
BG5	0.007607603	0.001331605	0.001874578	0.000292463	0.001418967
BG6	0.006394825	0.001751236	0.003434336	0.000247366	0.001037167
BG7	0.010627233	0.001197206	0.0004224	0.000235352	0.000129311
BG8	0.011488814	0.001622988	0.00069932	0.000263046	0.000233068
BG9	0.013336799	0.001785075	0.00078801	0.000184686	0.000203204
BG10	0.010922909	0.002031606	0.002711088	0.000338978	0.000925819
BG11	0.008123463	0.001715279	0.002577591	0.0004233	0.001527441
BG12	0.008301572	0.001339045	0.001941181	0.000262531	0.001217326

BG13	0.008402536	0.001896583	0.002909706	0.00020755	0.000920957
BG14	0.012692434	0.001623488	0.000548648	0.000269103	0.000141836
BG15	0.008174522	0.001697732	0.002896759	0.000317391	0.00171166
SZ1	0.006728153	0.00143095	0.00209129	0.000562898	0.000753768
SZ2	0.010170252	0.001245036	0.000385063	0.000575721	0.000166357
SZ3	0.007800236	0.001824265	0.002105975	0.000517084	0.000834936
SZ4	0.009004384	0.001863534	0.002464237	0.000488537	0.00086892
SZ5	0.00809357	0.001497373	0.002466456	0.000823861	0.001786326
SZ6	0.006738604	0.001689971	0.003571189	0.000586816	0.001282432
SZ7	0.010899722	0.001229605	0.000384263	0.000771721	0.000104924
SZ8	0.011718029	0.001731054	0.000607853	0.000443635	0.000302723
SZ9	0.012472702	0.001695774	0.000676852	0.001215091	0.000161729
SZ10	0.007872976	0.001517854	0.002009517	0.000976291	0.000618277
SZ11	0.007850281	0.00170762	0.002646213	0.000538511	0.001771324
SZ12	0.008926141	0.001483584	0.002344885	0.000488823	0.001743218
SZ13	0.00857191	0.00193401	0.002943534	0.000359132	0.001039665

	4	9			
SZ14	0.013485214	0.001768071	0.000577089	0.000641378	0.000163648
SZ15	0.008206164	0.001774732	0.0030977	0.000461099	0.002251681
TG1	0.00462512	0.000426083	0.001327432	0.001992463	0.000711616
TG2	0.007922043	0.000416305	0.000409989	0.002443768	0.000163477
TG3	0.006824652	0.00066457	0.001969345	0.001632171	0.001023053
TG4	0.006790721	0.000583496	0.00210195	0.001767059	0.001115148
TG5	0.0062647	0.000490432	0.002104066	0.002148315	0.001915922
TG6	0.004389844	0.000491282	0.002598163	0.001683753	0.00143207
TG7	0.008856735	0.000440261	0.000395045	0.002109063	0.000208081
TG8	0.008091301	0.000478579	0.000678629	0.002772032	0.000405401
TG9	0.012068093	0.000678328	0.000731734	0.002443323	0.000229509
TG10	0.007004763	0.000565169	0.001888601	0.002255256	0.000768901
TG11	0.005004041	0.000476463	0.001857102	0.00236585	0.002012253
TG12	0.006859343	0.000470342	0.001780547	0.001904462	0.001658005
TG13	0.006535499	0.000611256	0.002329022	0.001269799	0.000937165

TG14	0.007894392	0.000443418	0.00046584	0.003256583	0.000193109
TG15	0.007064293	0.000605576	0.002257865	0.000863789	0.001586648

Table S11 Volatile organic compounds (VOCs) with corresponding retention times, Drift time and peak volumes

Count	Compound	Rt /s	Dt [a.u.]	Peak volume S	Peak volume BG	Peak volume SZ	Peak volume TG
1	gamma-Butyrolactone	1198.657	1.08525	646.219795	794.9307106	1231.535167	2410.972989
2	(E)-2-octen-1-ol	1189.052	1.15454	312.5216433	480.6886233	915.8448644	1184.431576
3	5-methyl furfural	1116.79	1.12918	415.0084283	612.9731694	974.0093906	2766.309894
4	methyl 2-furoate	1046.924	1.47639	74.56733039	74.48055644	77.81586989	415.0137417
5	Acetic acid	944.36	1.05139	8811.024361	10913.08711	11659.90411	13022.81656
6	Methional	968.98	1.08704	51.26036461	70.54781906	113.5058775	245.2138539
7	1H-indene	969.499	1.11836	64.68247333	69.49788761	103.4024195	313.5961417
8	1-acetyloxy-2-propanone	927.91	1.10029	725.9815939	1284.159356	2221.789756	2994.511767
9	1-Octen-3-ol	918.541	1.15988	2408.020817	4569.791822	7751.962511	11565.64217
10	1,2-Propanediol-diacetate	911.335	1.22134	86.27333439	244.2213189	837.097665	1559.856394
11	2-furaldehyde	900.9	1.08	137.227	188.385	1157.56	4282.05

		86	302	2094	9917	9473	475
12	(E)-2-octenal	905.067	1.33309	135.6845278	484.5926056	1649.093772	2595.596217
13	2,3-diethylpyrazine	851.135	1.22686	628.341215	672.7403667	661.4509589	646.4553067
14	3,4,5-Trimethoxytoluene	841.013	1.41825	63.95519944	68.78567339	85.33399978	202.595735
15	5-methyl-2(3H)-Furanone	838.116	1.12183	118.8871144	83.231546	99.61718622	416.492115
16	2-Ethyl-3-methylpyrazine	789.999	1.16553	42.77809039	33.50781361	37.101353	115.0294258
17	Cinnamyl acetate	784.867	1.08243	48.89820433	46.35126372	60.36141378	345.5780611
18	2-ethyl-6-methylpyrazine	772.094	1.17293	13.49024531	14.73890539	17.76835056	139.2244992
19	2-ethyl-5-methylpyrazine	765.777	1.19996	647.5901511	237.306165	223.6123783	237.0628783
20	hexyl formate	738.178	1.32682	342.1452361	502.7811217	1219.503541	5452.460889
21	2,5-Dimethylpyrazine	700.818	1.11576	56.50989672	77.15414072	127.8737392	649.4310578
22	2,6-Dimethylpyrazine	693.772	1.13636	20.86605486	24.93333461	44.92819039	357.6137639
23	Indole	700.293	1.14864	136.4127931	274.6356278	238.7837978	187.9381717
24	ethyl hex-3-enoate	689.275	1.254	16.74878433	18.91505417	25.91118	95.88009389
25	(E,E)-2,4-Hexadienal	712.475	1.45037	20.78767389	22.23703833	35.69716561	248.5734844
26	allyl isothiocyanate	695.237	1.36144	27.91229556	29.65666733	41.99095094	267.8083583

27	beta-Elemene	688.375	1.43754	24.54204828	31.54446944	48.28486039	132.9084956
28	2-Phenylethanol-propanoate	681.834	1.34547	1404.04505	2802.633583	4447.419389	3923.7147
29	Undecanal	688.6	1.59604	125.7456111	369.5757706	1404.805783	2573.617872
30	anisole	664.364	1.05376	138.3273817	561.4788983	3603.493194	6495.320494
31	2-Ethylpyrazine	661.703	1.15261	63.17447811	113.0566997	255.1017978	626.6295522
32	linarodin	661.836	1.22827	770.213875	1273.623274	1988.141911	2123.3326
33	Diphenyl oxide	659.574	1.29484	47.67645306	87.53631217	187.0273494	390.3991139
34	3-hydroxy-2-Butanone	640.339	1.05905	60.61396017	111.9020975	208.4861372	427.5498411
35	Cyclohexanone	638.861	1.15647	19.05515206	25.49693517	64.91786344	203.2875817
36	5,6,7,8-tetrahydroquinoxaline	638.801	1.20253	18.77149983	27.4179415	76.99096117	268.0558406
37	menthyl acetate	638.151	1.23353	188.3189644	319.5718383	1019.932197	3276.495433
38	2-Hexanol	637.382	1.27958	27.8249035	34.92150494	52.16785272	100.8881893
39	p-Cymen-7-ol	637.5	1.33095	967.2297633	1348.729642	2604.890667	8032.638183
40	butyl pentanoate	645.425	1.40446	151.3596844	136.8946813	249.2671833	962.1615461
41	nonanoic acid	636.554	1.55059	77.26239144	55.92741122	57.67709072	535.5253711
42	2,6-Dimethylpyridi	610.3	1.08	313.071	313.888	547.771	7560.54

	ne	12	506	6706	6844	5272	9228
43	(E)-2-Hexenal	604.207	1.18362	2230.815994	1550.895939	1657.897939	1656.695333
44	Dihydrocarveol	601.218	1.31945	88.80830206	51.05336517	60.03135078	220.9486639
45	3-methylbutyl ester Butanoic acid	602.926	1.38782	218.426785	129.8119014	140.8943167	147.1293517
46	Pentyl ester hexanoic acid	593.531	1.53805	27.2551315	27.83255667	24.7093015	84.40305994
47	1-Pentanol	577.045	1.51083	593.2433167	734.6036078	627.9095683	639.0102417
48	Isoquinoline	560.356	1.19785	59.17138522	53.87741556	52.42558289	213.9285956
49	1-Dodecene	560.144	1.42767	45.42748211	28.42738589	31.23020589	105.5828853
50	Cyclohexylamine	518.466	1.18161	2848.219828	1669.150711	1654.938728	1603.77415
51	hexyl acetate	515.266	1.41251	43.43574761	41.51943239	48.2372145	90.7110405
52	Pyridine	496.542	1.24553	1119.783428	381.1280983	386.8721061	370.2034306
53	Veratrole	510.698	1.57719	73.11315194	100.5308481	219.8740478	546.8026844
54	2-(2-Butoxyethoxy) ethanol	496.391	1.31447	24.86445828	25.75972633	35.35031544	143.934008
55	3-methyl-1-Butanol	496.09	1.48758	1004.579838	981.3739589	1144.123411	1920.449639
56	2,3-dihydrobenzofuran	488.286	1.09952	391.1688567	608.674195	697.14775	466.9774317
57	Cyclopentanone	464.498	1.10542	44.66120239	52.66492183	77.38471583	259.7181078

58	2-pentylfuran	458.595	1.25283	476.5781517	502.7559444	596.79686	831.1185183
59	Isophorone oxide	463.63	1.33342	356.7867456	216.6862367	249.0385883	254.2570089
60	Butyl hexanoate	458.074	1.46019	116.8927896	111.6363433	101.5830002	366.6137139
61	Linalooloxide (pyranoid)	449.218	1.26266	2909.815428	2619.6233	2549.276539	1851.059733
62	(E,Z)-2,6-Nonadien-1-ol	429.018	1.17707	74.61966628	92.07869111	101.5532517	157.8182578
63	3-Penten-2-one	410.355	1.0685	196.5246228	213.3874606	236.27734	282.8289406
64	ethyl (E)-2-butenoate	402.117	1.18372	2615.127311	1797.187006	1649.101417	1298.523639
65	Isophorone	399.426	1.25795	62.17083472	80.92209294	116.8250234	185.38185
66	Phenethyl formate	397.913	1.28564	1872.566528	1989.091372	1959.281139	1760.71135
67	Citronellal	401.539	1.38148	271.6130922	284.097225	278.1547361	436.9736717
68	gamma-Heptalactone	399.791	1.52859	1157.222739	1309.443511	1422.599672	1911.866117
69	Ethylbenzene	386.987	1.07545	99.78715578	78.73175211	132.0760553	275.928225
70	Naphthalene	385.389	1.11559	125.3914792	80.47982767	63.69006333	90.53095017
71	2-Methoxy-4-methylphenol	382.421	1.1827	263.2557344	178.2162467	236.4485439	643.78925
72	Allyl sulfide	383.505	1.3268	161.6050922	170.3126322	217.1531906	315.9833611
73	Tetramethylpyrazin	371.9	1.21	1890.65	2183.53	2277.74	1963.89

	e	76	099	3561	9072	0122	5989
74	Methyl benzoate	350.484	1.20802	508.9765294	510.2161739	574.0427639	857.35691
75	1-Penten-3-ol	350.292	1.3501	515.6706161	428.5283022	497.7101978	497.2623889
76	5-methyl-2-thiophenecarboxaldehyde	340.522	1.17322	151.9078561	249.8665811	338.2877961	420.9116739
77	(E,E)-2,4-Octadienal	335.906	1.26507	252.3627906	516.4266494	603.3549233	506.5651244
78	(E)-2-pentenal	335.665	1.36123	40.80783456	121.3324686	472.052675	1060.364181
79	Nonanal	338.119	1.47357	19.16599606	18.155934	25.57321711	106.2156081
80	3-Hydroxy-2-methyl-4H-pyran-4-one	333.975	1.5257	127.8430063	151.6493878	529.5204194	1410.36995
81	1,4-Dichlorobenzene	320.21	1.12863	226.4300122	73.47666639	95.68087228	180.2221778
82	Acetic acid butylester	313.317	1.23729	58.06973461	40.46345278	76.60288328	242.3296911
83	N-Nitrosodi-N-propylamine	313.571	1.27185	96.36691817	94.60082256	143.1551372	734.0330806
84	ethyl trans-2-hexenoate	313.238	1.32145	829.0860444	343.5374456	418.2959806	564.5319133
85	1-Phenylethanone	305.504	1.18015	790.1476217	581.237365	668.0814617	737.6300806
86	dipropyl disulphide	304.854	1.4701	37.22157783	49.30800617	148.6736344	195.8700494
87	furaneol	299.815	1.20106	800.4250089	570.9228417	778.6688639	1576.899194
88	3-Methyl-2(5H)-furanone	290.017	1.11146	766.4699839	709.1485194	743.6521961	666.4545989

89	n-butylcyclohexane	289.74	1.25385	1364.582289	2927.709839	3758.844617	3683.927611
90	6-Methyl-5-hepten-2-one	272.672	1.18078	1348.545491	1965.878072	2370.607783	2284.024772
91	2-methyl-1-propyl acetate	270.427	1.23355	13537.9125	9763.276333	7496.040444	8696.080028
92	N-Methylpyrrolidone	272.251	1.10727	210.1763167	412.5185228	828.9594017	649.35291
93	trimethylpyrazine	247.986	1.1695	101.9244181	130.4910384	258.1284022	462.932615
94	Myrcene	252.746	1.28843	152.2187867	208.0862089	308.0053156	615.3907433
95	2-Formylpyrrole	250.427	1.37804	62.12664578	106.2537492	212.6041472	217.2884756
96	Ethyl acetoacetate	223.818	1.14913	563.3178183	1013.566561	1259.975894	920.2926778
97	5-Methyl-3-heptanone	227.724	1.27784	1894.3086	2875.965467	3566.9124	5209.312606
98	S-propyl thioacetate	195.714	1.19775	207.8848883	246.0535778	355.7383011	680.5960772
99	3-Heptanone	197.659	1.22475	281.6824789	280.9429844	353.0896528	530.1303044
100	(E)-3-Hexen-1-ol	182.104	1.23896	33.51262744	19.84081056	24.88642967	51.89321133
101	ethyl 2-hydroxypropanoate	166.938	1.13878	453.2599478	64.93292228	93.12627689	156.1890472
102	Pentyl formate	170.438	1.28089	156.10425	120.7239382	132.1260486	119.9824673
103	4-Methyl-3-penten-2-one	157.346	1.12244	51.35750744	51.01954411	50.71478517	87.57507172

104	Propanoic acid	147.883	1.10041	125.8943488	154.998895	140.7761894	135.8293206
